# Neuropsychological Diagnostic Tools in Mild Cognitive Impairment: A Systematic Review and Meta-Analysis

**DOI:** 10.14336/AD.2025.0618

**Published:** 2026-08-20

**Authors:** Nuria Jerez-Garrido, Ana M. Jiménez-García, David Sánchez-Borda, Natalia Arias

**Affiliations:** ^1^BRABE Group, Department of Psychology, Faculty of Life and Natural Sciences, University of Nebrija, C/del Hostal, 28248 Madrid, Spain.; ^2^INEUROPA, Instituto de Neurociencias del Principado de Asturias, Oviedo, Spain.; ^3^ISPA, Instituto de Investigación Sanitaria del Principado de Asturias, Oviedo, Spain.

**Keywords:** Mild Cognitive Impairment, Alzheimer’s disease;, meta-analysis, cognitive assessment, neuropsychological tests

## Abstract

Due to baseline differences in cognitive test performance and their variable capacity to predict progression from Mild Cognitive Impairment (MCI) to Alzheimer’s disease (AD), this systematic review and meta-analysis aimed to identify the most frequently used neuropsychological tests and their predictive value. Given the high prevalence of MCI, identifying accurate diagnostic tools is crucial. This study examined cognitive test differences between MCI patients and healthy individuals in clinical studies. A random-effects meta-analysis was conducted to account for variability. To this end, a comprehensive search was conducted across PubMed, Scopus, ScienceDirect, and Cochrane databases. Of the 103 studies that met the inclusion criteria and were reviewed, 34 were excluded from the meta-analysis due to the absence of control groups, and 7 articles were excluded because there were not enough comparable studies to perform a meaningful analysis. The meta-analysis revealed significant cognitive impairment in global cognition, language, and emotional well-being. Some studies reported hippocampal atrophy and amyloid-β/tau deposition in MCI patients progressing to AD, while others found benefits from physical and cognitive training. These findings highlight the need for standardized cognitive assessments to identify MCI patients at risk of AD conversion and implement effective treatments. Nevertheless, this meta-analysis faced limitations due to missing patient data, including the MCI subtype and disease duration.

## INTRODUCTION

According to the World Health Organization (www.who.int/news-room/fact-sheets/detail/dementia) approximately 50 million people worldwide are affected by dementia, a number expected to triple by 2050. Decades ago, distinguishing dementia from typical cognitive aging was sufficient. However, the growing need to identify preclinical stages for early intervention has expanded the spectrum to include Mild Cognitive Impairment (MCI) [1]. MCI is broadly defined as a transitional stage between normal aging and various forms of dementia, including but not limited to Alzheimer’s disease (AD) [2,3]. Clinically, however, it has often been treated as a prodromal stage of probable AD [4]. During this preclinical phase, some patients experience progressive neuronal and axonal loss that usually results in episodic memory impairment and medial temporal lobe atrophy [5]. However, not all individuals with MCI, even those on the AD trajectory, present with memory deficits or hippocampal atrophy [6,7], suggesting the involvement of alternative or non-amnestic pathological processes. These patients, despite cognitive changes, do not yet meet the clinical criteria for dementia [6,7]. Consequently, MCI is a highly prevalent condition among older adults. Although over 50% of individuals with MCI will progress to AD within 4 to 6 years [8], it is important to emphasize that not all cases evolve into dementia. Given its high prevalence, the increased risk of conversion to AD, and the challenge of predicting which individuals will deteriorate, it is crucial to determine whether any neuropsychological test or psychometric inventory can reliably forecast this phenoconversion.

The 5^th^ edition of the Diagnostic and Statistical Manual of Mental Disorders (DSM-5) provides a broad framework for defining dementia from a clinical perspective but does not specify which neuropsychological tests should be used for diagnosis. As a result, there is a lack of standardized diagnostic tools [9] across these conditions, including MCI. Epidemiological studies underscore its increasing impact. In 2018, approximately 17% of individuals aged 65 and older were estimated to have MCI [10]. By 2020, the number of people living with MCI in the United States was twice that of individuals clinically diagnosed with AD, with projections suggesting this figure could nearly double again by 2060 [11]. More recently, in 2022, MCI was estimated to affect 22% of individuals aged 65 and older.

Alarmingly, despite the high prevalence of MCI, it is estimated that only 8% of Americans with the condition have received a formal diagnosis [12]. This diagnostic gap is concerning, as MCI is characterized by preserved independence in basic daily functions but mild difficulties with instrumental activities, such as shopping, paying bills, and cooking, which can significantly impact quality of life [13]. Patient independence often complicates a challenge for early diagnosis, which remains crucial for effective management and intervention. According to Petersen’s criteria [14], a diagnosis of MCI requires: (a) subjective complaints of memory impairment, (b) difficulties with normal daily activities, (c) preserved general cognitive function, (d) objectively impaired memory function relative to age, and (e) absence of dementia.

Alternatively, the National Institute on Aging-Alzheimer’s Association (NIA-AA) criteria define MCI based on: (a) concern about a change in cognition, (b) impairment in one or more cognitive domains, (c) preserved independence in functional abilities, and (d) absence of dementia [15]. In contrast to Petersen’s framework, the NIA-AA criteria also incorporate biomarkers to increase diagnostic specificity and to identify cases more likely associated with Alzheimer’s pathology [16]. This biomarker-based classification enables clinicians to estimate the likelihood that MCI reflects an underlying AD process [17].

As previously mentioned, MCI is generally considered an transitional phase between normal aging and dementia disorders, particularly AD [18]. Indeed, individuals with MCI face an increased risk of developing AD and some already exhibit amyloid-β deposition, a neuropathological hallmark of AD. Studies utilizing PET imaging to assess AD biomarkers have shown that approximately half of individuals with MCI display brain changes associated with AD [19]. However, MCI does not inevitably progress to dementia. While it may represent the earliest cognitive stage of AD, it can also result from other conditions, including neurological, systemic, or psychiatric disorders [20]. Indeed, the literature presents various classifications of MCI, but the most widely accepted distinction is between amnestic (aMCI) and non-amnestic (naMCI) MCI. aMCI is characterized by memory impairment and carries a higher risk of phenoconversion to AD. In contrast, naMCI preserves memory function while impairing other cognitive domains, such as language, visuospatial abilities, or executive function [21]. Although naMCI is not a strong risk factor for AD, it may lead to other forms of dementia. Ultimately, the probability of progression to dementia is estimated to be 49% for aMCI patients and 27% for those with naMCI [6].

In this context, early diagnosis of MCI is particularly relevant, as it can help slow progression to dementia and AD by enabling timely multi-domain lifestyle interventions and/or the initiation of pharmacological treatments during the early stages of the disease. A formal cognitive evaluation is essential for diagnosing MCI; however, while the DSM-5 does not provide specific guidelines for MCI identification nor any dementia diagnosis, the Montreal Cognitive Assessment (MoCA) and the Mini-Mental State Examination (MMSE) are among the most widely used tools in clinical practice [10]. Given the clinical heterogeneity and dynamic nature of MCI, this systematic review and meta-analysis aimed to identify the most frequently used neuropsychological tests in the assessment of MCI. The current study explored how these tests capture cognitive variation, providing insight into which tools are most effective for monitoring the neuropsychological trajectory of MCI. These findings offer valuable guidance for selecting sensitive and reliable assessments in both research and clinical practice.

## MATERIALS AND METHODS

### Research strategy

A systematic bibliographic search was conducted in accordance with Preferred Reporting Items for Systematic Reviews and Meta-Analysis (PRISMA) guidelines, as detailed in Supplementary Materials (Supplementary Table 1). Separate searches for original articles related to MCI were conducted across four electronic databases: PubMed, Scopus, ScienceDirect and Cochrane. The search was performed in January 2025 by two researchers (DS, NJ). The database search terms are provided in the Supplementary Material. No chronological or methodological filters were applied, except for title, keyword, and abstract. All resulting datasets were then exported and organized in Mendeley and Excel.

### Study selection

After removing duplicates, all remaining titles and abstracts were screened for eligibility. Epidemiological studies and articles that did not focus specifically on assessing MCI in patients or their relationship with cognitive deficits were considered ineligible. Following the initial selection, full texts were retrieved and reviewed based on the inclusion criteria. For a study to be included, it had to: (1) involve experimental cases related to MCI, (2) show measurements of MCI in the experimental subjects, and (3) examine cognitive changes in these subjects.

The inclusion and exclusion criteria were structured using the PICOS framework (Population, Intervention, Comparison, Outcomes, and Study Design). The population included individuals diagnosed with Mild Cognitive Impairment (MCI) and cognitively healthy control subjects. The ‘intervention’ component referred to the presence of MCI identified by neuropsychological evaluation. Comparisons were made between individuals with MCI and those without the condition. Outcomes focused on performance in neuropsychological tests, and eligible study designs included observational studies with cross-sectional or longitudinal data.

### Data extraction

The following data were systematically extracted from each study meeting the inclusion criteria: study characteristics (country of origin, study design, and participant recruitment methods); sample information (total number of participants, group classification—e.g., MCI vs. control—and sex distribution); and clinical data (comorbidities, current treatments or interventions, and duration of the condition, when reported). We also collected neuropsychological data, including the tests administered and scores, as well as sociodemographic characteristics such as age and educational level. When specified, we documented the MCI subtype and any available neuroimaging or brain-related findings.

### Methods quality

The methodological quality of the clinical research studies was assessed using the Newcastle-Ottawa Scale (NOS) [22]. This scale evaluates three key domains: selection of study groups (4 points maximum), comparability of cohorts (2 points maximum), and assessment of outcomes (3 points maximum), allowing a comprehensive appraisal of methodological rigor in observational and quasi-experimental studies. The maximum score is 9 points; higher scores indicate better quality. Based on the predefined grading system (7-9 = low risk of bias, 4-6 = moderate risk, <4 = high risk), the overall quality of the studies included in this systematic review was high: the median NOS score was 9 (mean = 8.2; range = 4-9), and 88.9% of studies scored ≥7 points. The most frequent score was 9 (55.6% of studies), followed by 8 (24.2%) and 7 (9.1%). Only a small proportion of studies fell into the moderate-quality range (11.1%), and none were classified as high risk of bias. The bar chart showing the score distribution is presented in Figure 1.

**Figure 1.**
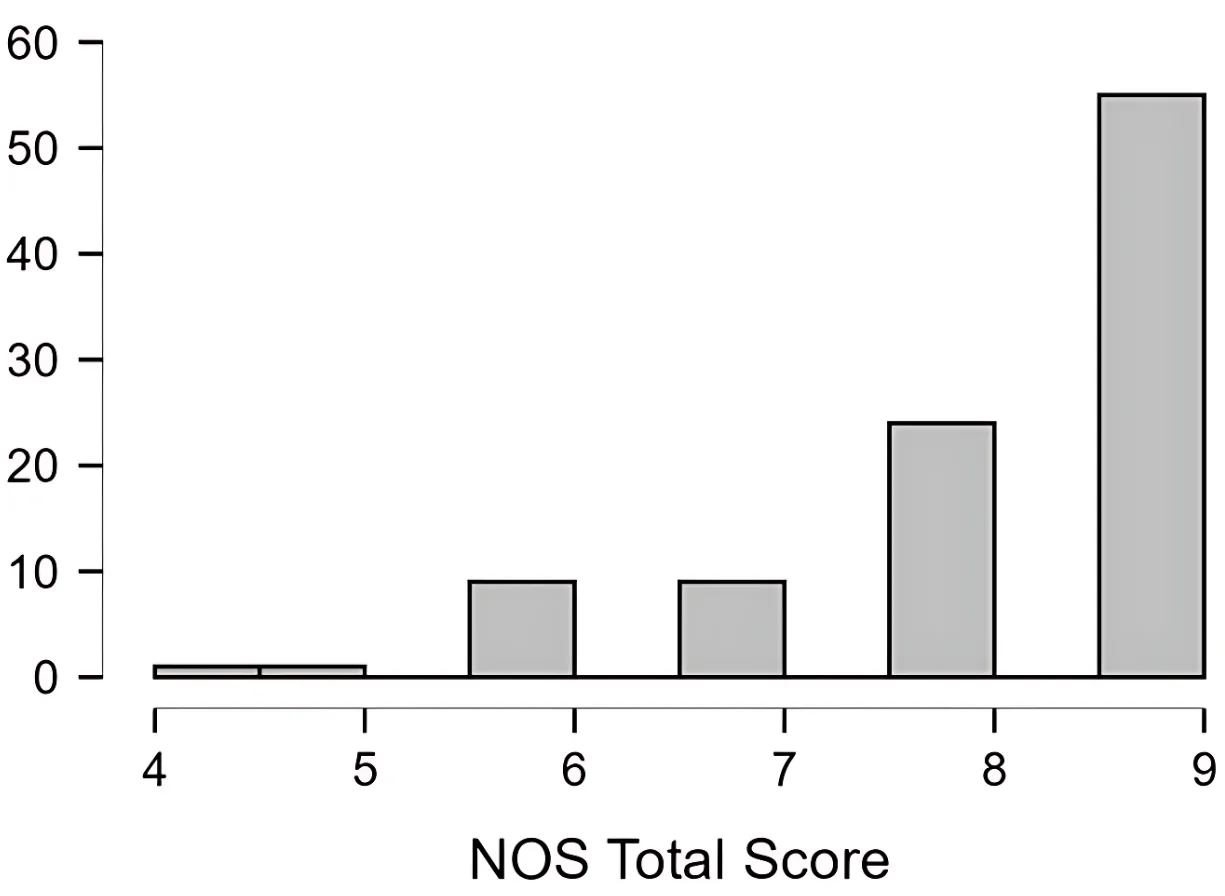
Bar chart showing the frequency of studies (Y-axis) according to the NOS score (X-axis).

### Meta-analysis

A meta-analysis is a statistical synthesis of data from original research, identified through a systematic review and selected based on predefined inclusion criteria [23]. In the present study, we employed a random-effects meta-analysis, which assumes that the true effect size may vary across studies due to differences in populations, settings, or methodologies. This contrasts with fixed-effect models, which assume a single underlying effect size [24].

Accordingly, we conducted a continuous random-effects meta-analysis using the standard mean difference (SMD) to examine cognitive changes and assess the predictive and stratifying value of neuropsychological tests, psychometric tools, inventories, and scales in relation to Mild Cognitive Impairment (MCI). This analysis compared cognitive changes in healthy adults and MCI patients. Studies reporting assessments of (i) Global Cognition, including the Mini-Mental State Examination (MMSE), Montreal Cognitive Assessment (MoCA), and the Clinical Dementia Rating (CDR) (ii) Attentional processes with the Trail Making Test (TMT) Part A and B; (iii) Language, evaluated with the Verbal Frequency Test, Letter Fluency, Boston Naming Test, and the California Verbal Learning Test (CVLT-II); and (iv) Instrumental Activities of Daily Living (IADL), which assess the ability to perform more complex daily tasks required for independent living, such as managing finances, using transportation, shopping, and handling medication; (vi) Mood and Emotional Well-being, using the Hospital Anxiety and Depression Scale (HADS), Hamilton Depression Rating Scale (HAM-D), and the Geriatric Depression Scale (GDS), were included in the analysis. For outcomes with fewer than 10 studies (k < 10), Hedges’ t-test was applied for robustness. A mixed-effects meta-regression was also conducted to assess the influence of a selected covariate. Significance did not influence study inclusion, and null findings were included.

A total of 60 studies were included in the meta-analysis. Of the 103 studies included in this systematic review, 32 articles were excluded from the meta-analysis due to lack of a control or comparable group, and 7 due to insufficient comparable studies. Authors of relevant publications were contacted for raw datasets. Means, standard deviations (SDs), and sample sizes were entered into R Studio software version 4.3.1, which calculated the SMD, confidence intervals (CIs), heterogeneity, and overall effect size for cognitive changes. The ‘metafor’ package in R Studio was used for these calculations [25].

**Figure 2.**
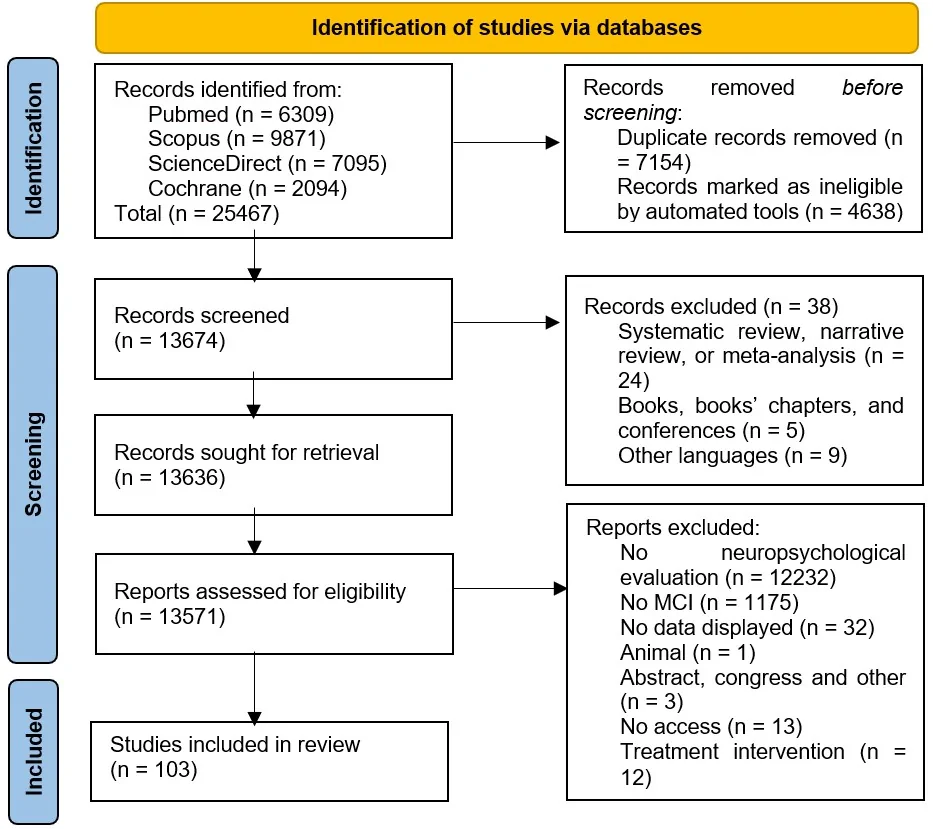
**PRISMA flow chart of selection of publications for inclusion in review**. PRISMA, Preferred Reporting Items for Systematic Reviews and Meta-Analysis.

In the heterogeneity analysis, Cochran's *Q* statistic and the *I^2^* index were used to assess variability among studies. For analyses with more than two studies, the Egger test was applied, while the Begg and Mazumdar's rank correlation test was also used to evaluate potential publication bias. The Egger’s test detects funnel plot asymmetry, identifying potential small-study effects or selective reporting[26].

To further address heterogeneity, we performed leave-one-out sensitivity analyses, recalculating the meta-analysis each time excluding one study to identify influential cases (outliers). The Fail-Safe N, based on Rosenthal’s approach, was also calculated using the 'metafor' package. Sub-analyses were performed when high variability was observed. Forest plots were generated using Review Manager (RevMan) to visually represent effect sizes and CIs.

## RESULTS

### The search and screening process is illustrated in Figure 2.

#### General characteristics of selected studies

The studies selected for inclusion in the systematic review were published between 2002 and 2023 (n = 103) and involved human participants, totaling 12,848 individuals, both with and without MCI, for comparative purposes. The mean age of participants was 73.85 ± 7.85 years, with approximately 54.83% women and 45.17% men.

Regarding study design, the sample included 24 transversal (non-randomized observational) studies, 22 cross-sectional studies, 19 longitudinal studies, 6 cohort studies, 5 clinical trials, 5 experimental studies, 4 prospective cohort studies, 4 longitudinal observational studies, 4 case reports, 3 prospective studies, 2 prospective case-control studies, 2 retrospective studies, 2 validation studies, 1 comparative cross-sectional study, and 1 exploratory study. Only 19.42% of the studies specified the MCI subtype of participants, while the remaining 80.58% referred to a general MCI diagnosis without further classification.

Additionally, 71 studies (61.73%) included a control group of cognitively healthy individuals [27-96], whereas 32 did not [2,5,97-127].

In terms of geographic distribution, 37.86% of studies were conducted in the United States. Another 31.07% originated from European countries—primarily Greece, France, Germany, and the Netherlands, with smaller contributions from the United Kingdom, Hungary, Norway, Poland, Finland, Portugal, Spain, Austria and Sweden. A total of 17.48% of studies were conducted in Asia (Japan, South Korea, Taiwan, and China), and the remainder came from Australia (7.77%), Brazil (2.91%) and Canada (2.91%).

From these 103 studies, global cognition was assessed using the Mini-Mental State Examination (MMSE) in 75 articles, the Clinical Dementia Rating (CDR) in 14, the Montreal Cognitive Assessment (MoCA) in 11, and the Alzheimer’s Disease Assessment Scale-Cognitive Subscale (ADAS-Cog) in 6, among other less frequently used tests.

Tests assessing episodic memory and learning included the Rey Auditory Verbal Learning Test (RAVLT) and the Free and Cued Selective Reminding Test (FCSRT) in 5 articles each; the California Verbal Learning Test-II (CVLT-II) in 4; the Wechsler Memory Scale (WMS) Logical Memory Immediate and Delayed subtests in 3; and the Hopkins Verbal Learning Test (HVLT) in 3. Attention and executive functions were evaluated using the Trail Making Test A (TMT-A) and B (TMT-B), both in 12 articles, and the Digit Span subtest from the Wechsler Adult Intelligence Scale-Third Edition (WAIS-III) in 10. Language was assessed using the Verbal Fluency Test (VFT) in 15 studies and the Boston Naming Test (BNT) in 8.

Emotional state was primarily measured using the Geriatric Depression Scale (GDS) in 28 articles, the Hamilton Depression Rating Scale (HAM-D) in 6, and the Hospital Anxiety and Depression Scale - Depression subscale (HADS-D) in 5; anxiety was assessed using the HADS - Anxiety subscale (HADS-A) in 5.

Functional status and daily activities were evaluated in 4 studies using the Instrumental Activities of Daily Living (IADL) scale. Dementia was assessed with the Consortium to Establish a Registry for Alzheimer’s Disease (CERAD) in 4 articles, the Dementia Rating Scale (DRS) in 4, and the Hachinski Ischemic Score (HIS) in 3. A comprehensive list of all tests used in the included articles and their frequency of appearance is presented in Table 1.

**Table 1:** Overview of the neuropsychological tests used to assess cognitive domains in the studies included in this review, along with the number of studies in which each test was applied.

Cognitive aspect measured	Test	Number of appearances in articles
**Global cognition**	Mini-Mental State Examination (MMSE)	75
	**Clinical Dementia Rating (CDR)**	14
	**Montreal Cognitive Assessment (moca)**	11
	**Alzheimer's Disease Assessment Scale-Cognitive Subscale (ADAS Cog), also including:** -ADAS Cog13 -ADAS Cog11	6 2 1
	**Cognitive Abilities Screening Instrument (CASI)**	2
	**Modified Mini-Mental State Examination (3MS)**	1
**Episodic memory and learning**	Rey Auditory Verbal Learning Test (RAVLT)	5
	**Free and Cued Selective Reminding Test (FCSRT)**	5
	**California Verbal Learning Test-II (CVLT-II)**	4
	**Wechsler Memory Scale (WMS) Logical Memory Immediate and Delayed**	3
	**Hopkins Verbal Learning Test (HVLT)**	3
	**Rey-Osterrieth Complex Figure Test (ROCFT)**	2
	**Rey-Osterrieth Complex Figure Test - Delayed Recall (RFDR)**	2
	**Wechsler Memory Scale - Fourth Edition (WMS-IV)**	1
**Attention and executive functions**	Trail Making Test A (TMT A)	12
	**Trail Making Test B (TMT B)**	12
	**Wechsler Adult Intelligence Scale (WAIS) III Digit Span**	10
	**Stroop color and word test**	2
	**Wisconsin Card Sorting Test (WCST)**	1
	**Symbol Digit Modalities Test (SDM)**	1
	**Wechsler Memory for Auditory Encoding (WMAE)**	1
	**Modified Card Sorting Test (MCST)**	1
**Language tests**	Verbal Fluency Test (VFT)	15
	**Boston Naming Test (BNT) also including:** -BNT-30	8 1
	**Deno100**	1
**Visual memory and visuospatial skills**	Brief Visuospatial Memory Test (BVMT)	1
	**Benton Visual Retention Test (BVRT)**	1
**Depression state**	Geriatric Depression Scale (GDS)	23
	**Hamilton Depression Rating Scale (HAM-D)**	6
	**Hospital Anxiety and Depression Scale - Depression (HADS-D)**	5
**Anxiety state**	Hospital Anxiety and Depression Scale - Anxiety (HADS-A)	5
	**Hamilton Anxiety Rating Scale (HAM-A)**	2
	**State-Trait Anxiety Inventory (STAI)**	1
**Functional daily activities**	Instrumental Activities of Daily Living (IADL)	4
	**Activities of Daily Living (ADL)**	2
	**Functional Activities Questionnaire (FAQ)**	2
	**Everyday Cognition Scale (ecog)**	2
	**Mild Behavioral Impairment Checklist (MBI)**	1
	**Apathy Evaluation Scale - Behavioral Subscale (ABS)**	1
	**Functional Rating Scale for Symptoms of Dementia (FRSSD)**	1
	**Informant-based Subjective Memory Decline (I-SMD)**	1
	**Rivermead Behavioural Memory Test (RBMT)**	1
	**Functional Cognitive Assessment Scale (FUCAS)**	1
**Dementia**	Consortium to Establish a Registry for Alzheimer’s Disease (CERAD)	4
	**Dementia Rating Scale (DRS)**	4
	**Hachinski Ischemic Score (HIS)**	3
	**Addenbrooke's Cognitive Examination (ACE)**	1
	**Mattis Dementia Rating Scale (MDRS)**	1
**Cognitive decline**	Informant Questionnaire on Cognitive Decline in the Elderly (IQCODE)	1
**Neuropsychiatric symptoms**	Neuropsychiatric Inventory (NPI)	2
**Other supplementary tests**	Cambridge Cognitive Examination (CAMCOG)	2
	**Auditory Verbal Learning Test (AVLT)**	1
	**Royal Prince Alfred Prospective Memory Test (RPA-promem**	1
	**National Adult Reading Test (NART)**	1
	**Wide Range Achievement Test - Fourth Edition (WRAT-IV)**	1

#### Cognitive differences

All of the studies included determined cognitive differences using standard cognitive tests for humans. The most used test was MMSE (n = 75), but others such CDR (n = 14) and GDS as emotional evaluation tool (n = 23) were also employed. All three emphasized significant differences with lower scores in MMSE but higher scores in CDR and GDS in MCI patients. Among the tests included, the Mini-Mental State Examination (MMSE), Clinical Dementia Rating (CDR), and Montreal Cognitive Assessment (MoCA) were most frequently used as diagnostic tools for identifying MCI. Other tests were used either for cognitive screening in cross-sectional designs or to evaluate cognitive change in longitudinal studies. To maintain methodological consistency and reduce bias when comparing studies with different designs, only baseline scores were included in the meta-analysis. Main neuropsychological tests organized by cognitive domain are represented in Table 2. A detailed description of all the features is provided in Supplementary Table 2.

**Table 2:** Description of key neuropsychological tests organized by cognitive domain.

Source	Cognitive profile	Cognitive changes
**Global cognition**		
**Bernstein et al. [79]**	MCI; Controls	MCI patients displayed lower results in MoCA.
**Bidzan et al. [11]**	MCI stable; MCI converting	MCI stable patients displayed lower scores in MMSE than MCI converting patients.
**Bowen et al. [100]**	MCI	Cognitive decline (MoCA) correlated with lower extremity strength and increased sedentary behavior.
**Boyle et al. [55]**	MCI; Controls	Controls exhibited higher scores in MMSE than MCI patients.
**Bregman et al. [97]**	Underestimators; Overestimators	Overestimators scored higher in MMSE than underestimators.
**Buerger et al. [85]**	MCI; Controls	MCI patients displayed lower in MMSE test than healthy controls.
**Burggren et al. [86]**	aMCI; Controls	Healthy controls performed better in MMSE than aMCI.
**Capp et al. [64]**	aMCI; Controls	Controls displayed better results in MMSE.
**Cardoso et al. [69]**	aMCI; Controls	aMCI patients scored lower in MMSE.
**Chan et al. [105]**	Stable MCI; Converters to dementia	The group that ended in dementia scored lower in MMSE.
**Chen et al. [106]**	Stable aMCI; Converters	The stable group obtained higher scores in MMSE.
**Cook et al. [70]**	aMCI; Controls	Control subjects scored higher in MMSE.
**De Anna et al. [77]**	Controls; aMCI - better DMS48; aMCI - worse DMS48	Controls exhibited higher results in MMSE.
**Devanand et al. [72]**	MCI; Controls	Healthy controls scored higher in MMSE.
**Ewers et al. [71]**	MCI; Controls	Healthy controls displayed better results in MMSE than MCI patients.
**Fodor et al. [64]**	MCI; Controls	MCI subjects displayed higher results in ADAS Cog13 than controls
**Forstmeier et al. [63]**	MCI; Controls	Healthy controls scored higher in MMSE than MCI patients.
**Gao et al. [62]**	MCI; Controls	MCI patients scored lower on MMSE and MoCA compared to healthy controls.
**Ghoshal et al. [61]**	MCI; Controls	Controls scored higher in MMSE than MCI patients.
**Grundman et al. [78]**	MCI; Controls	Healthy controls scored higher in MMSE and HIS than MCI patients.
**Hanyu et al. [111]**	Early MCI; Late MCI without hypoperfusion; Late MCI with hypoperfusion	LMCI- patients scored the lowest in MMSE and MoCA, followed by LMCI+.
**Headley et al. [59]**	MCI; Controls	MCI patients scored lower in MMSE.
**Johansson et al. [92]**	MCI; Controls	Healthy controls scored higher on MMSE but lower on ADAS-Cog compared to MCI patients.
**Jung et al. [81]**	MCI; Controls	In MMSE controls scored higher than MCI patients, but lower in CDR.
**Kinsella et al. [56]**	aMCI; Controls	aMCI patients scored lower than controls in MMSE.
**Kirsebom et al. [54]**	MCI; Controls	MCI patients exhibited lower scores in MMSE than controls.
**Kivisäkk et al. [53]**	aMCI; naMCI; Controls	Both, aMCI and naMCI scored lower in MMSE but higher in CDR than controls.
**Kuhn et al. [52]**	aMCI; Controls	aMCI patients exhibited lower scores in MMSE than healthy controls.
**Lai et al. [51]**	aMCI; Controls	aMCI patients showed lower scores in MMSE than healthy patients.
**Landau et al. [80]**	Early MCI; Late MCI; Controls	Controls scored higher on MMSE but lower on ADAS-Cog than both MCI groups; early MCI also outperformed late MCI on both measures.
**Lazarou et al. [50]**	MCI; Controls	Healthy controls exhibited better results in MMSE tests than MCI patients.
**Lazarou et al. [49]**	MCI; Controls	MCI patients scored lower in MMSE than healthy controls.
**Lim et al. [48]**	PiB+ Controls; PiB- Controls; PiB+ MCI; PiB- MCI	MCI patients had higher CDR scores than controls, regardless of PiB status, while PiB+ MCI patients had lower MMSE scores, though not significantly different from PiB- MCI.
**Lin et al. [113]**	aMCI; naMCI; Controls	naMCI patients scored higher in MMSE than aMCI patients.
**Lopes et al. [46]**	MCI; Controls	MCI patients scored lower in MMSE.
**MacAulay et al. [45]**	aMCI; Controls	Healthy controls scored higher in MMSE test than MCI patients.
**Montero-Odasso et al. [90]**	aMCI; naMCI; Controls	Controls scored highest on both MMSE and MoCA, followed by the naMCI group, while the aMCI group obtained the lowest scores.
**Moscoso et al. [81]**	Early MCI; Late MCI; Controls	Healthy controls scored higher on MMSE and lower on CDR than MCI patients. Among MCI groups, early MCI performed better on MMSE but worse on CDR.
**Park [42]**	MCI; Controls	MCI patients exhibited lower results in MMSE and CDR when compared to controls.
**Richard et al. [118]**	MCI without apathy or depressed affect; MCI with apathy only; MCI with depressed affect only; MCI with both apathy and depressed affect.	MCI patients without apathy or depressed affect scored the highest in MMSE, followed by those with only apathy, only depressed affect, and lastly, those with both symptoms.
**Riemenschneider et al. [108]**	Progressive MCI; Stable MCI	Progressive MCI patients scored lower in MMSE.
**Rodakowski et al. [38]**	aMCI; Controls	MCI patients displayed lower in MMSE than controls.
**Schreiber et al. [120]**	MCI Aβ-; MCI Aβ+	MCI Aβ- patients had lower ADAS Cog score but higher RAVLT score than MCI Aβ+ patients.
**Small et al. [89]**	aMCI; Controls	Healthy controls and aMCI patients obtained similar results in MMSE.
**Stewart et al. [96]**	MCI; Controls	Healthy controls scored higher than MCI patients in MMSE.
**Taler et al. [37]**	a MCI; Controls	aMCI patients exhibited lower scores than healthy controls in MoCA.
**Thomas et al. [36]**	MCI; Controls	Healthy controls showed higher results in MMSE than MCI patients.
**Toth et al. [35]**	MCI; Controls	MCI patients exhibited lower results in MMSE but higher results in the ADAS Cog than healthy controls.
**Traykov et al. [88]**	MCI; Controls	Healthy controls scored higher in MMSE.
**Troyer and Murphy [34]**	aMCI; Controls	Patients with aMCI displayed lower scores in MMSE.
**Troyer et al. [33]**	aMCI; Controls	Healthy controls scored higher than aMCI patients on MMSE, HVLT-R immediate recall, WAIS III digit symbol coding, and TMT B, while aMCI patients scored higher on WAIS III digit span.
**Tsunoda et al. [32]**	MCI; Controls	MCI portrayed lower MMSE.
**Van der Flier et al. [31]**	MCI; Controls	MCI patients had lower scores in the MMSE than healthy controls.
**Van Patten et al. [30]**	MCI; Controls	aMCI and naMCI were assembled in a single group and performed MMSE with lower results than healthy patients.
**Visser et al. [29]**	aMCI; naMCI; Controls	naMCI patients presented lower scores in MMSE than controls. However, aMCI patients obtained the worst results.
**Wolk et al. [28]**	MCI; Controls	aMCI showed lower scores in MMSE than healthy controls.
**Wei et al. [125]**	Early MCI; Late MCI; Controls	Early MCI patients had higher scores in MMSE and MoCA; but lower scores in CDR than late MCI patients.
**Xie et al. [27]**	aMCI; Controls	MMSE showed that aMCI had lower score than healthy controls.
**Xu et al. [103]**	Low CRP MCI; High CRP MCI	MCI patients with low plasma CRP obtained higher results than MCI patients with high plasma CRP in MMSE test.
**Yokoi et al. [102]**	MCI converters to AD; MCI non-converters to AD	MCI patients that converted to AD had higher scores in ADAS-Cog and CDR, but lower scores in MMSE than non-converters.
**Zhang et al. [98]**	MCI Mahjong players; MCI non-players	Significant differences were found in MoCA, with significant improvements in the MCI patients who played Mahjong.
**Zhu et al. [99]**	MCI	MCI patients scored low in MMSE and MoCA.
**Attentional processes**		
**Fodor et al. [64]**	MCI; Controls	Healthy controls scored lower in TMT A and TMT B than MCI patients.
**Gao et al. [62]**	MCI; Controls	MCI patients displayed higher scores in TMT A and TMT B than healthy controls.
**Hansen et al. [5]**	aMCI (longitudinal data); aMCI (clinical settings)	aMCI patients detected in longitudinal data displayed higher results in TMT B, but lower in TMT A than aMCI patients detected in clinical settings.
**Kirsebom et al. [54]**	MCI; Controls	MCI displayed lower results than controls in TMT A and TMT B.
**Lazarou et al. [49]**	MCI; Controls	MCI patients scored lower in TMT B than healthy controls.
**Park [42]**	aMCI + Program; aMCI - Program	aMCI who participated in the program scored lower in TMT A and Digit Span than the aMCI group who did not participate.
**Small et al. [89]**	aMCI; Controls	In TMT A and TMT B, aMCI patients displayed higher results.
**Traykov et al. [88]**	MCI; Controls	Healthy controls scored lower in in TMT A and TMT B than MCI.
**Troyer and Murphy [33]**	aMCI; Controls	Patients with aMCI displayed lower scores in TMT B than healthy controls. Whereas in WAIS III Digit Span aMCI scored higher.
**Troyer et al. [34]**	aMCI; Controls	Healthy controls scored higher in TMT B than aMCI patients. Nevertheless, these scored higher in WAIS III digit span.
**Wei et al. [125]**	Early MCI; Late MCI	Early MCI patients scored higher in RAVLT learning but lower in RAVLT immediate, RAVLT-perc-forgetting, TMT A, and TMT B compared to late MCI patients.
**Zhu et al. [99]**	MCI	MCI patients scored low in RAVLT delayed recall, but high in TMT A and B.
**MEMORY**		
**Cardoso et al. [69]**	aMCI; Controls	aMCI patients scored lower across in WMS logical memory immediate and WMS logical memory delay.
**De Anna et al. [77]**	Controls; aMCI - better DMS48; aMCI - worse DMS48	Controls exhibited higher results in FCSRT delayed and total recall.
**Devanand et al. [72]**	MCI; Controls	Healthy controls scored lower in FCSRT.
**Fodor et al. [64]**	MCI; Controls	Healthy controls scored higher in RAVLT delayed recall than MCI patients.
**Kinsella et al. [56]**	aMCI; Controls	aMCI patients scored lower than controls on all tests, including HVLT, WMS logical memory, and WMS IV verbal paired associates.
**Taler et al. [37]**	aMCI; Controls	aMCI patients exhibited lower scores than healthy controls in WMS-R immediate and delayed.
**Traykov et al. [88]**	MCI; Controls	Healthy controls scored higher in FCSRT free recall, total, free delayed recall and WAIS III digit symbol encoding.
**Troyer et al. [33]**	aMCI; Controls	Healthy controls scored higher in HVLT-R immediate recall.
**Xie et al. [27]**	aMCI; Controls	WMS Logical Memory Immediate and WMS Logical Memory Delayed showed that aMCI had lower score than healthy controls.
**Language**		
**Chan et al. [105]**	Stable MCI; Progressive MCI	The group that ended in dementia scored lower in VFT.
**Gao et al. [62]**	MCI; Controls	MCI patients displayed lower scores in BNT, Digit Span and VFT-Animals than healthy controls.
**Hansen et al. [5]**	aMCI - longitudinal data; aMCI - clinical settings	aMCI patients detected in longitudinal data displayed higher results in VFT-Animals and BNT than aMCI patients detected in clinical settings.
**Lazarou et al. [49]**	MCI; Controls	MCI patients scored lower in VFT than healthy controls.
**Metzler-Baddeley et al. [43]**	aMCI; Controls	Healthy controls scored higher in VFT than aMCI patients.
**Small et al. [89]**	aMCI; Controls	Controls scored higher in BNT and VFT-Animals.
**Segkouli et al. [67]**	aMCI; Controls	aMCI patients scored lower in BNT than controls.
**Taler et al. [37]**	aMCI; Controls	aMCI patients exhibited lower scores than healthy controls in BNT, Letter Fluency and VFT-Animals.
**Traykov et al. [88]**	MCI; Controls	Healthy controls scored higher in VFT.
**Wei et al. [125]**	Early MCI; Late MCI	Early MCI patients had higher scores in BNT and VFT than late MCI patients.
**Weitzner and Calamia [84]**	MCI; Controls	Healthy controls scored higher in Category Fluency and Letter Fluency.
**Wolk et al. [28]**	aMCI; Controls	aMCI showed lower scores in VFT-Animals and BNT-30 than healthy controls.
**Zhu et al. [99]**	MCI	MCI patients scored low in BNT.
**Activities in daily life**		
**Bregman et al. [97]**	Underestimators; Overestimators	Overestimators scored higher in ECogSubject but lower in ECogStudyPartner than underestimators
**De Anna et al. [77]**	Controls; aMCI - better DMS48; aMCI - worse DMS48	Controls exhibited higher results in IADL.
**Jung et al. [57]**	MCI; Controls	Controls scored lower in IADL than MCI patients.
**Rodakowski et al. [38]**	aMCI; Controls	MCI patients displayed higher scores in IADL test.
**Mood and emotional well-being**		
**Bregman et al. [97]**	Underestimators; Overestimators	GDS scores were higher in overestimators than in underestimators.
**Burggren et al. [86]**	aMCI; Controls	Healthy controls performed lower in HAM-D than aMCI.
**Faria et al. [65]**	Stable MCI; Progressing to dementia	The MCI stable group scored higher in GDS than the MCI patients who ended in dementia.
**Grundman et al. [78]**	MCI; Controls	Healthy controls scored lower in CDR and HAM-D than MCI patients.
**Hanyu et al. [111]**	Early MCI; LMCI-; LMCI+	Early MCI patients scored lowest in GDS-15, while LMCI- scored the highest.
**Headley et al. [59]**	MCI.	MCI patients scored higher in CDR.
**Johansson et al. [92]**	MCI; Controls	Controls scored lower in HADS-D and HADS-A than MCI patients.
**Jung et al. [57]**	MCI; Controls	Controls scored lower in GDS than MCI patients.
**Kirsebom et al. [54]**	MCI; Controls	MCI displayed lower results than controls in CERAD learning and CERAD recall.
**Lim et al. [48]**	PiB+ controls; PiB- controls; PiB+ MCI; PiB- MCI	MCI patients scored higher in HADS-D and HADS-A than controls independently of their PiB (a ligand for labelling amyloid).
**Lin et al. [113]**	aMCI; naMCI	naMCI patients scored higher in HAM-D and HADS-D than aMCI patients.
**Maeng et al. [44]**	MCI; Controls	MCI patients exhibited higher results in GDS than controls.
**Polcher et al. [40]**	MCI; Controls	MCI patients had lower scores in GDS than controls but lower in CERAD.
**Rabin et al. [39]**	AMCI; NaMCI; Controls	aMCI and naMCI scored lower in GDS than healthy patients.
**Riemenschneider et al. [19]**	Progressive MCI; Stable MCI	Progressive MCI patients scored lower across all tests, including CERAD delayed verbal recall and GDS.
**Rodakowski et al. [38]**	aMCI; Controls	MCI patients displayed higher scores in HAM-D than controls.
**Thomas et al. [36]**	MCI; Controls	MCI patients scored higher in GDS.
**Tsunoda et al. [32]**	MCI; Controls	MCI portrayed higher CDR.
**Wolk et al. [28]**	aMCI; Controls	aMCI showed lower scores in CERAD immediate total, CERAD delayed recall, CERAD delayed recognition, than healthy controls.
**Xie et al. [27]**	aMCI; Controls	HAM-Anxiety and GDS showed that aMCI had lower score than healthy controls.
**Yokoi et al. [102]**	Converters to AD; Non-converters	MCI patients that converted to AD had higher scores in GDS.

#### Meta-analysis

A total of 60 studies were included in the present meta-analysis. The analyses allowed evaluation of differences between healthy adults and individuals with MCI across multiple dimensions, including global cognition, memory, attention, language, daily living activities, mood and emotional well-being.

##### A) Global cognition

The evaluation of global cognition was conducted using the Mini-Mental State Examination (MMSE), the Montreal Cognitive Assessment (MoCA), and the Clinical Dementia Rating (CDR) scale.

###### A.1) Mini-Mental State Examination (MMSE)

A total of *k*=60 studies were included in the meta-analysis of global cognition with the Mini-Mental State Examination (MMSE). The overall estimated effect size was -1.09 (95% CI: -1.27 to -0.90), which was statistically significant (*Z*=-11.5, *p*<0.001), indicating differences between individuals with MCI and healthy controls, with MCI patients scoring lower than healthy controls. The analysis revealed very high heterogeneity (*I²*=93.87%, *τ²*=0.4708), confirmed by a highly significant heterogeneity test(*Q_(59)_*=583.217, *p*<0.001), suggesting substantial variability beyond chance. Publication bias assessments yielded mixed results. The fail-safe N was substantial (33,404), indicating that many null studies would be required to negate the observed effect. However, Egger’s regression test (*t*=-3.412, p<0.001) suggested potential funnel plot asymmetry, whereas Begg and Mazumdar’s rank correlation test (*p*=0.179) did not indicate significant publication bias.

**Figure 4.**
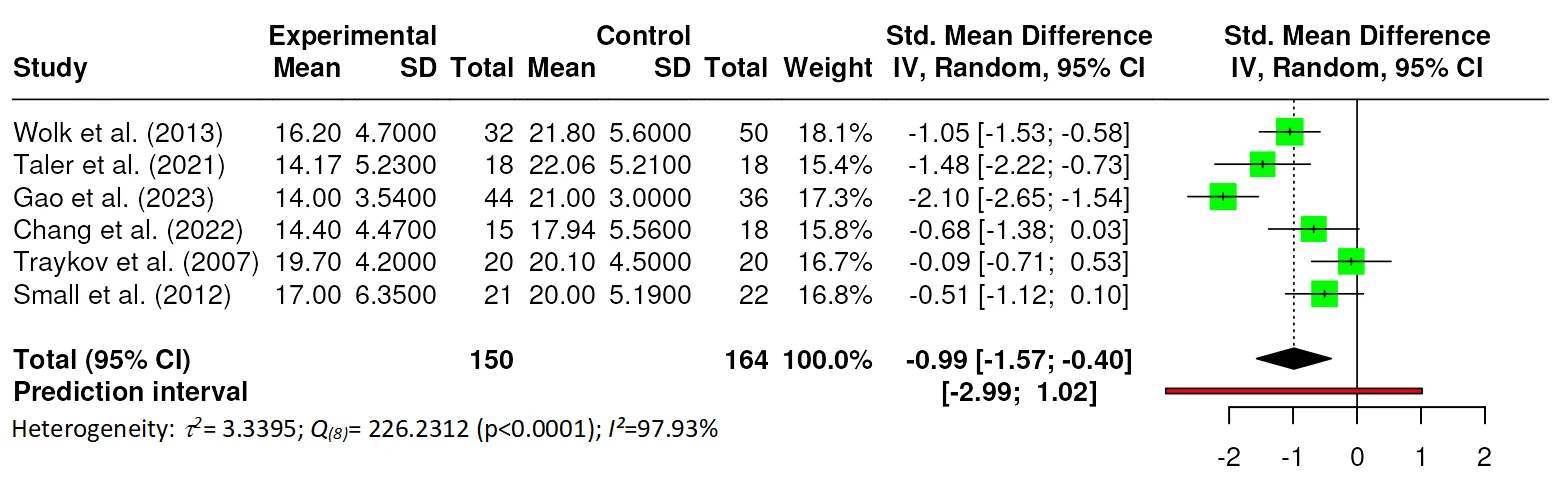
**Meta-analysis using a random effects model of selected studies assessing verbal fluency through the “Animals in 1 Minute Test” in Alzheimer's disease (AD) patients**. The plot displays the effect estimates and corresponding confidence intervals (CIs) for each study included in the meta-analysis. The relative weight or contribution of each study to the overall effect estimate is also provided in percentages. The overall weighted effect is indicated by a diamond at the bottom of each subfigure. The figure was generated using R software version 4.3.1.

Sensitivity analyses identified one potential outlier [62], with a studentized residual exceeding ±3.34. Excluding this study slightly reduced heterogeneity (*I²* = 92.01%) and yielded a pooled estimate of -1.0491 (95% CI: -1.2325 to -0.8657, *p* < 0.0001). Moderator analyses indicated that neither the biomarker presence as an inclusion criterion nor MCI subtype (amnestic vs. non-amnestic) significantly influenced effect size. Specifically, biomarker presence showed a borderline but non-significant association (*p* = 0.055), accounting for only 5.31% of variability, while MCI subtype showed no significant association (*p* = 0.196).

###### A.2) Montreal Cognitive Assessment (MoCA)

A total of *k*=5 studies were included in the meta-analysis assessing global cognition with the Montreal Cognitive Assessment (MoCA). The overall estimated effect size was -2.3643 (95% CI: -3.7567 to -0.9718), statistically significant (*t*(_4)_ = -3.2846, *p* = 0.0304) (Fig. 3), indicating differences between individuals with MCI and healthy controls, with MCI patients scoring lower. However, heterogeneity was extremely high (*I²*=95.54%, *τ²*=2.3989, *Q_(4)_*=56.8985, p<0.0001), reflecting substantial variability. The funnel plot showed no clear asymmetry, and Egger’s regression test (*t*=-1.9937, *p*=0.1402) did not detect statistically significant publication bias, although limited sample size may affect test power.

Sensitivity analyses revealed Gao et al. [62] strongly influenced the the pooled effect and accounted for most heterogeneity: removing this study markedly decreased heterogeneity (*I²* = 40.78%, τ² = 0.0676) and changed the effect size estimate to -1.6809 (95% CI: -2.2993 to -1.0626, *p* = 0.0032). Subsequently, a mixed-effects meta-regression examined whether MCI subtype (amnestic vs. non-amnestic) moderated the effect size.

###### A.3) Clinical Dementia Rating (CDR)

A total of *k*=9 studies were included in the meta-analysis assessing global cognition with the Clinical Dementia Rating (CDR). The overall estimated effect size was 2.9586 (95% CI: 0.5204 to 5.3968), statistically significant ( *t_(8)_* = 2.7982, *p* = 0.0233) (Fig. 3B), indicating differences between individuals with MCI and healthy controls, with MCI patients scoring higher.The analysis revealed extremely high heterogeneity, (*τ²*=10.7476 and *I²*=99.59%), indicating nearly all variability is due to between-study differences rather than sampling error. The heterogeneity test was highly significant (*Q_(8)_*=431.7337 p<0.0001), confirming substantial variation. The funnel plot and Egger’s test (*t*=2.1205, *p*=0.0668) provided borderline evidence for publication bias, suggesting a possible small-study effect but no strong confirmation. Sensitivity analysis showed that the overall effect remained large and significant regardless of study removal. However, excluding Jung et al. [57] notably reduced heterogeneity (*I²* = 98.91%) and yielded a smaller pooled effect (g = 2.2556, 95% CI: 0.8168 to 3.6945), indicating substantial influence on effect magnitude and variability. Additionally, Park [42] and Moscoso et al. [81] (both comparisons A and B) moderately influenced effect size and heterogeneity when excluded, though they were not statistical outliers. A mixed-effects meta-regression found biomarker presence as an inclusion criterion did not moderateeffect size *(p* = 0.9005), with *R²* = 0% indicating no explanation of heterogeneity.

##### B) Attentional processes

A total of *k* = 6 studies were included in the meta-analysis, assessing processing speed using the Trail Making Test - Part A (TMT-A) under a random-effects model (REML). The pooled estimated effect size was *g* = 0.8593 (95% CI: -0.7123 to 2.4309), not statistically significant (*t*_(5)_ = 1.4055, *p* = 0.2189), indicating no reliable performance differences between individuals with MCI and healthy controls. The analysis revealed very high heterogeneity (*τ²* = 2.1163, SE = 1.3909; *I²* = 96.95%), indicating most variability is due to between-study differences rather than sampling error. The heterogeneity test was highly significant (*Q*_(5)_ = 90.7290, *p* < 0.0001), confirming substantial variation. Funnel plot inspection and Egger’s test (*p* = 0.5148), with the Begg and Mazumdar rank correlation test (*p* = 0.4694), revealed no publication bias. Sensitivity analysis showed removing any single study did not change overall significance; however, Gao et al. [62] had the greatest influence, and its exclusion markedly reduced heterogeneity (*I²* = 88.34%, *τ²* = 0.4543) while maintaining a non-significant pooled effect.

A total of *k* = 9 studies were included in the meta-analysis examining global executive function using the Trail Making Test - Part B (TMT-B) under a random-effects model (REML). The pooled estimated effect size was *g* = 0.8017 (95% CI: -0.4827 to 2.0861), not statistically significant (*t*(8) = 1.4393, *p* = 0.1880), indicating no robust performance differences between individuals with MCI and healthy controls. The analysis revealed very high heterogeneity (*τ²* = 2.6533, SE = 1.3715; *I²* = 97.39%), suggesting nearly all variability is due to between-study differences. The heterogeneity test was highly significant (*Q*_(8)_ = 191.2511, *p* < 0.0001), confirming substantial variation. Funnel plot inspection and Egger’s test (*p* = 0.3935), with the Begg and Mazumdar rank correlation test (*p* = 0.7614), provided no publication bias. Sensitivity analysis indicated no single study altered overall significance; however, Gao et al. [62] had the highest influence, and its removal reduced heterogeneity (*I²* = 94.87%) and *τ²* to 1.2188, although the pooled effect remained non-significant.

##### C) Language

Language and verbal abilities were assessed using various tests targeting different linguistic and cognitive domains. Verbal fluency, verbal memory, verbal and lexical access and naming skills.

###### C.1) Verbal Fluency Test

Verbal fluency was evaluated through the Animals in 1 Minute Test which assesses semantic and phonological fluency, providing insights into lexical access and executive control.

A total of *k*=6 studies were included in the meta-analysis of verbal fluency, assessed using the Animals in 1 Minute Test, which measures semantic fluency by the ability to rapidly generate words within a category. The overall estimated effect size was -0.9901 (95% CI: -1.5750 to -0.4051), statistically significant ( *t*_(5)_ = -3.34, *p* = 0.0206; Fig. 4), indicating lower scores in individuals with MCI compared to healthy controls. The analysis revealed substantial heterogeneity (*I²*=81.98%, *τ²*=0.4342), as confirmed by a significant heterogeneity test (*Q_(5)_* = 28.2445, *p*<0.0001), suggesting variability is primarily due to study differences rather than random error.

**Figure 5.**
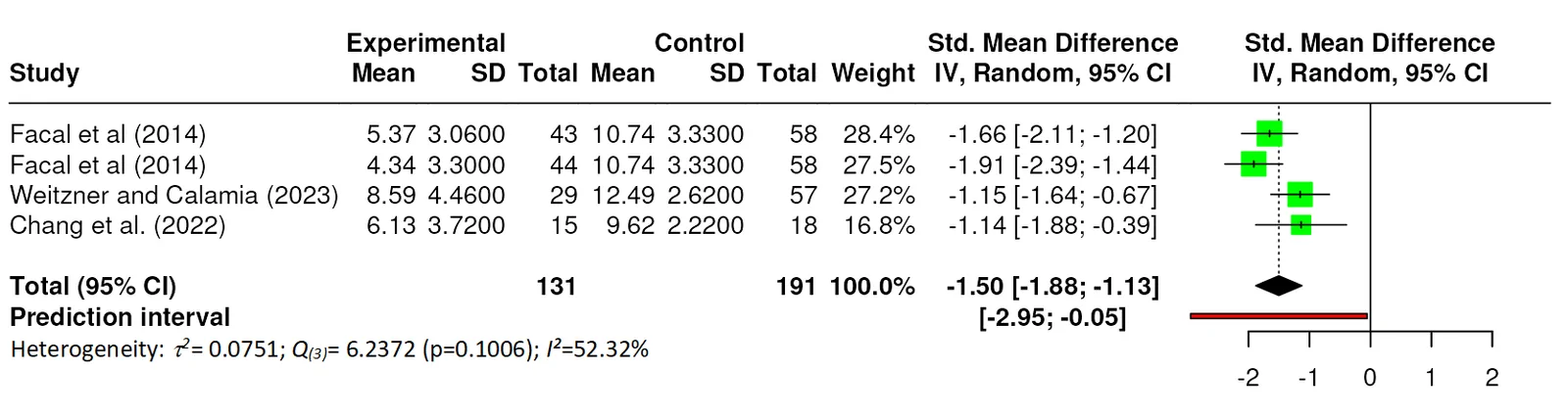
**Meta-analysis using a random effects model of selected studies assessing verbal learning and memory though the California Verbal Learning Test (CVLT-II) - Long-delay free recall in Alzheimer's disease (AD) patients**. The relative weight or contribution of each study to the overall effect estimate is also provided in percentages. The overall weighted effect is indicated by a diamond at the bottom of each subfigure. The figure was generated using R software version 4.3.1.

Sensitivity analyses identified Gao et al. [62] as influential, with a high studentized residual (-2.46). Removing this study reduced heterogeneity from 81.98% to 61.50% and changed the pooled effect size to -0.7549 (95% CI: -1.3994 to -0.1105, *p* = 0.0313), although effect direction and significance remained. The funnel plot and both Egger’s regression test (*p* = 0.7523) and Begg and Mazumdar’s rank correlation test (*p* = 1.0000) showed no significant publication bias.

###### C.2) Verbal Memory

Verbal memory was assessed using the California Verbal Learning Test (CVLT-II), including the Long-Delay Free Recall (LDFR) subscale, which measures episodic memory and the capacity to retain and recall verbal information over varying time intervals.

A total of *k*=4 studies were included in the meta-analysis of verbal memory using the CVLT-II. The overall estimated effect size was -0.0834 (95% CI: -1.8974 to 1.7307), not statistically significant (*t*_(3)_ = -0.0906, *p* = 0.9335), indicating no difference in scores between individuals with MCI and healthy controls. The analysis revealed very high heterogeneity (*I²*=97.93%, *τ²*=3.3395), confirmed by the significant heterogeneity test (*Q_(3)_*=226.2312, *p*<0.0001).

Sensitivity analyses identified Kinsella et al. [56] as highly influential, with an extreme studentized residual (17.89) and the largest Cook’s distance in the set. Removing this study eliminated heterogeneity (I² = 0%) and changed the pooled effect size to -1.0767 (95% CI: -1.7128 to -0.4406, p = 0.0183), revealing a statistically significant difference between groups. However, removing the other studies individually did not alter the non-significant results, indicating that the overall null finding is largely driven by the inclusion of Kinsella et al. [56].

The regression test for funnel plot asymmetry (p = 0.2600) and Begg and Mazumdar’s rank correlation test (p = 0.7500) showed no publication bias, and the funnel plot appeared symmetrical.

A total of *k*=4 studies were included in the meta-analysis of long-delay free recall, a CVLT-II subscale that evaluates memory retention after a longer interval. The overall estimated effect size was -1.5018 (95% CI: -1.8759 to -1.1277), highly significant (*t*_(3)_ = -7.92, *p* = 0.0042; Fig. 5), indicating significantly substantially lower scores in individuals with MCI compared to healthy controls. The analysis showed moderate heterogeneity (*I²*=52.32%, *τ²*= 0.0751, *Q_(3)_*=6.2372, *p*=0.1006), suggesting that part of the variability is due to differences between studies. The regression test for funnel plot asymmetry (*p* = 0.4736) and Begg and Mazumdar’s rank correlation test (*p* = 0.7180) did not indicate evidence of publication bias, and the funnel plot appeared symmetrical. Sensitivity analyses identified Facal et al. [73] and Weitzner & Calamia [84] as influential studies, with the former showing a strong negative influence (r_student_ = -1.60) and the latter a strong positive influence (r_student_ = 1.39). Removing either study reduced heterogeneity (*I²* down to 34.85% and 18.19%, respectively) and slightly modified the pooled effect size (range: -1.6585 to -1.3606), but the results remained statistically significant.

###### C.4) Verbal and lexical Access

Lexical access and naming skills were assessed using the Boston Naming Test (BNT), which evaluates the ability to retrieve and produce the names of visual stimuli, highlighting potential difficulties in lexical retrieval and language production. A total of *k*=6 studies were included in the meta-analysis of lexical access and naming ability, assessed using the Boston Naming Test (BNT). The overall estimated effect size was 0.8719 (95% CI: -0.0833 to 1.8271, *p*=0.0736), which was not statistically significant (*t*_(5)_ = 1.80, *p* = 0.1313), suggesting no meaningful difference between individuals with MCI and healthy controls. The analysis revealed very high heterogeneity (*I²*=92.45%, *τ²*=1.3029), confirmed by a highly significant test for heterogeneity (*Q_(5)_*=68.6347, *p*<0.0001). This indicates that nearly all observed stemmed from differences between studies rather than sampling error.

The Egger’s regression test (*p* = 0.8503) and Begg and Mazumdar’s rank correlation test (*p* = 0.6872) did not indicate significant publication bias, and the funnel plot appeared symmetrical. Sensitivity analyses showed that removing Gao et al. [62] substantially reduced heterogeneity (*I²* = 80.56%) and decreased the pooled effect size to 0.4546 (95% CI: -0.4830 to 1.3921, *p* = 0.2495), although the result remained non-significant. Influence diagnostics also identified Gao et al. [62] as an influential study (r_student_ = 2.93), exerting the strongest leverage on the overall model. While excluding this study attenuated the effect size and reduced heterogeneity, the overall conclusion of no statistically significant difference persisted.

##### D) Activities in daily life

Daily functioning was assessed through measures of Instrumental Activities of Daily Living (IADL), which evaluate complex tasks necessary for independent living such as managing finances, medication, and household responsibilities. A total of *k*=4 studies were included in the meta-analysis of daily living abilities, analyzed using a random-effects model with the REML estimator. The overall estimated effect size was 3.0485 (95% CI: -1.3711 to 7.4682), which was not statistically significant (*t*_(3)_ = 1.33, *p* = 0.2751), suggesting no meaningful difference in IADL performance between individuals with MCI and healthy controls. The analysis revealed extremely high heterogeneity (*I²*=99.48%, *τ²*=19.9655, *τ*=4.4683), confirmed by a significant heterogeneity test (*Q_(3)_*=67.9458, *p*<0.0001) suggesting that variability in effect sizes was mainly due to study differences rather than random error.

Influence diagnostics revealed that Jung et al. [57] was highly influential, with a studentized residual of 7.61 and a large impact on both heterogeneity (*I²* = 66.06%) and the pooled effect size (0.8910, 95% CI: -0.2365 to 2.0184, *p* = 0.0767) when removed. This suggests that the overall estimate is strongly driven by this study. The Egger’s regression test indicated potential funnel plot asymmetry (*t*_(2)_ = 4.73, *p* = 0.0420), although Begg and Mazumdar’s rank correlation test did not detect bias (*p* = 1.0000).

##### E) Mood and Emotional Well-being

The evaluation of mood and emotional well-being was conducted using different scales assessing depression and anxiety symptoms in individuals with MCI compared to healthy controls. The meta-analysis included the Hospital Anxiety and Depression Scale (HADS) and the Geriatric Depression Scale (GDS), including its short version (GDS-Short).

**Figure 6.**
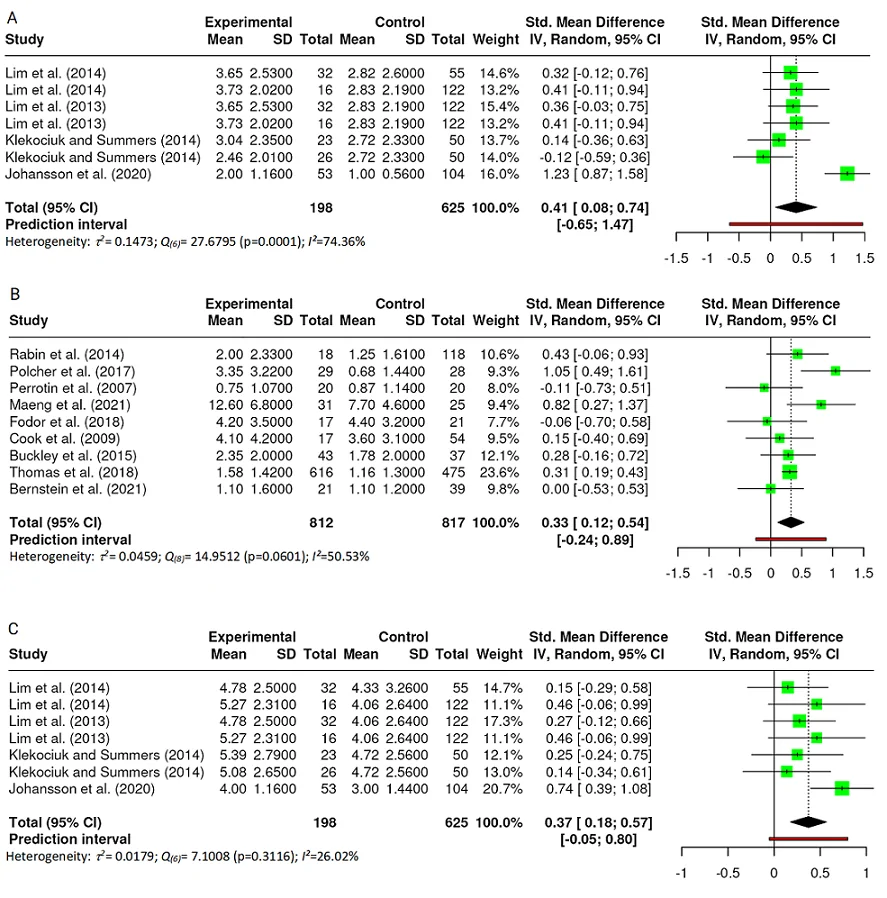
**Meta-analysis using a random effects model of selected studies assessing depression and anxiety scales in Alzheimer's disease (AD) patients**. (**A**) Hospital Anxiety and Depression Scale (HADS) - Depression Subscale, (B) Geriatric Depression Scale (GDS), (C) Hospital Anxiety and Depression Scale (HADS) - Anxiety Subscale. The relative weight or contribution of each study to the overall effect estimate is also provided in percentages. The overall weighted effect is indicated by a diamond at the bottom of each subfigure. The figure was generated using R software version 4.3.1.

A total of *k*=7 studies were included in the meta-analysis of depression symptoms, assessed using the Hospital Anxiety and Depression Scale (HADS-Depression Subscale). The overall estimated effect size was 0.4112 (95% CI: 0.0794 to 0.7430), which was statistically significant (*t*_(6)_ = 2.53, *p* = 0.0450; Fig. 6A), indicating higher levels of depressive symptoms in individuals with MCI compared to healthy controls. The analysis revealed substantial heterogeneity (*I²*=74.36%, *τ²*=0.1473), confirmed by a significant test for heterogeneity (*Q_(6)_*=27.6795, *p*=0.0001), suggesting variability beyond chance. Sensitivity analyses identified Johansson et al. [92] as highly influential; removing it reduced heterogeneity to 0% and decreased the pooled effect size to 0.2567 (95% CI: 0.0455 to 0.4680, *p* = 0.0261). Funnel plot inspection and Egger’s regression test did not indicate statistically significant asymmetry (*t*_(5)_ = -2.19, *p* = 0.0805), and Begg and Mazumdar’s rank correlation test was nonsignificant (*p* = 0.6411). A mixed-effects meta-regression tested whether biomarker inclusion criterio influenced the effect size. The moderator was not statistically significant (*F*(1, 5) = 0.0443, *p* = 0.8415), and the *R²* value was 0%, indicating no explanation of between-study variability.

A total of *k*=9 studies were included in the meta-analysis of geriatric depression, assessed using the Geriatric Depression Scale (GDS). The overall estimated effect size was 0.3639 (95% CI: 0.0678 to 0.6600), statistically significant (*t*_(8)_ = 2.76, *p* = 0.0247; Fig. 6B), indicating higher depression scores in individuals with MCI compared to healthy controls. The analysis detected moderate heterogeneity (*I²*=50.53%, *τ²*=0.0459), suggesting that just over half of total variability was due to differences between studies. The test for heterogeneity approached significance (*Q_(8)_*=14.9512, *p*=0.0601), indicating some inconsistency. Sensitivity analyses identified Polcher et al. [40] as most influential; excluding it reduced heterogeneity to nearly 0% and slightly decreased the pooled effect size to 0.2903 (95% CI: 0.1565 to 0.4240, *p* = 0.0014). The funnel plot and Egger’s test (*p*= 0.8975), and Begg and Mazumdar’s rank correlation test (*p* = 0.9195) indicated significant publication bias.

For the GDS-Short, *k*=6 studies were included. The overall estimated effect size was 0.2355 (95% CI: -0.2248 to 0.6959), not statistically significant (*t*_(5)_ = 1.01, *p* = 0.3599), suggesting no meaningful difference between groups. The analysis revealed substantial heterogeneity (*I²*=82.16%, *τ²*=0.2627), confirmed by a significant test (*Q_(5)_*=27.5951, *p*<0.0001). This indicates considerable variability beyond chance. Sensitivity analyses found no single study altering results or heterogeneity, and influence diagnostics did not identify any overly influential cases. Egger’s regression test indicated possible publication bias (*t*=6.2459, *p*=0.0033), with the intercept estimate (*b*=-1.6374, 95% CI: -2.3995 to -0.8752) suggesting asymmetry; however, Begg and Mazumdar’s test was not significant (p = 0.0556).

Finally, *k*=7 studies were included in the meta-analysis of anxiety symptoms, assessed using the HADS-Anxiety Subscale. The overall estimated effect size was 0.3731 (95% CI: 0.1784 to 0.5678), statistically significant (*t*_(6)_ = 4.20, *p* = 0.0057; Fig. 6C), indicating higher anxiety levels in individuals with MCI. The analysis revealed low to moderate heterogeneity (*I²*=26.02%, *τ²*=0.0179), and the test for heterogeneity was not significant (*Q_(6)_*=7.1008, *p*=0.3116), indicating limited variability beyond chance. Sensitivity analyses identified Johansson et al. [92] as most influential; removing it reduced heterogeneity to 0% and decreased the pooled effect size to 0.2749 (95% CI: 0.1341 to 0.4158, *p* = 0.0040). Funnel plot inspection, Egger’s regression test (*t*(5) = -1.27, *p* = 0.2604), and Begg and Mazumdar’s rank correlation test (*p* = 0.6411) did not indicate significant publication bias. A mixed-effects meta-regression model examined whether biomarker inclusion moderated the effect size. The moderator was not statistically significant (*p* = 0.5499), and the *R²* value was 0%, indicating it did not explain residual heterogeneity (*I²* = 25.97%).

## DISCUSSION

This systematic review and meta-analysis examined differences between healthy older adults and individuals with MCI across multiple cognitive and emotional domains using validated neuropsychological tests. The findings reveal significant impairments in global cognition, attention, memory, language, and emotional well-being, indicating that cognitive decline in MCI affects multiple domains, although test sensitivity varies considerably. Below, we discuss these results alongside relevant methodological considerations.

Significant differences emerged in global cognition measures (MMSE, MoCA, DRS, CAMCOG, and CDR), consistent with their design to capture broad cognitive changes. However, not all global cognition tools reliably detect these differences. Despite their widespread use to distinguish MCI from healthy controls, their limited sensitivity to early or subtle impairments likely contributes to observed variability. Indeed, high heterogeneity (*I²* > 90%) across studies for all global cognition measures suggests substantial variability in effect sizes. This heterogeneity likely reflects differences in sample characteristics (e.g., age, education, clinical subtypes), methodological factors (e.g., test administration, diagnostic procedures), and contextual variables such as study settings or instrument language. Notably, this meta-analysis used continuous data (means and standard deviations), but did not statistically model variability in diagnostic thresholds or cut-offs defining MCI across studies. Such discrepancies probably contributed to heterogeneity, as variations in operationalizing MCI and interpreting test scores may have influenced group classification and comparisons.

Additional heterogeneity sources stem from study design and implementation. The sample included approximately 45.6% cross-sectional, 36.9% longitudinal, and the remainder used other methodologies. Recruitment ranged widely from clinical populations and community samples to database cohorts. Diagnostic criteria inconsistently defined MCI subtypes and disease duration, and neuropsychological assessments varied markedly in administration. Some evaluations were conducted face-to-face by trained professionals, while others relied on telephone interviews, self-reports, or medical records, often with limited detail.

Tests like Digit Span (WAIS-III Forward and Backward) and the Trail Making Test (TMT-A and B) revealed impairments in working memory, sustained attention, and task-switching among MCI individuals. While TMT results were meta-analyzed, Digit Span findings were reviewed narratively due to limited data. Interestingly, TMT-B, which requires alternating between numerical and alphabetical sequences and places high demands on cognitive flexibility and divided attention, did not consistently show significant differences between groups [28,62,64,82,88-89]. Notably, executive function studies varied widely in methodology and showed substantial heterogeneity, suggesting these deficits may become more apparent in later MCI stages.

Common attention tests may lack ecological validity because real-world cognitive functioning often demands multitasking and integrative processing—abilities potentially compromised in MCI but not always captured by standard neuropsychological tools. Supporting this, neuroimaging studies report dorsolateral prefrontal cortex (DLPFC) [28-29] atrophy and altered activation during executive tasks, linking structural and functional changes to working memory and sustained attention deficits in MCI [110].

Episodic memory impairment remains the hallmark of MCI, confirmed by significant group differences in immediate recall. While analyses of WMS-Logical Memory [27,56,69] and the Free and Cued Selective Reminding Test (FCSRT) Total [77] and Delayed Recall [72,77] supported this pattern, the number of eligible studies was insufficient for meta-analytic synthesis. For example, the Total Recall analysis relied on two studies from the same research group [77], both of which scored low on the Newcastle-Ottawa Scale (NOS), limiting the generalizability and reliability of the results. Although the Delayed Recall analysis included an additional study by Devanand et al. [73], the total number of studies remained below the inclusion threshold. These limitations necessitate cautious interpretation and highlight the need for further high-quality research.

Regarding language tests, verbal fluency and naming tasks such as Animals in 1 minute and Boston Naming Test revealed early linguistic impairments, consistent with evidence that verbal fluency deficits are among the earliest MCI and AD Haga clic o pulse aquí para escribir texto.markers [64,67]. These deficits ikely arise from prefrontal dysfunction, especially in the left DLPFC and inferior frontal gyrus, critical regions for word retrieval, lexical access, and cognitive flexibilityHaga clic o pulse aquí para escribir texto.[64,67]. Among these tests, 'Animals in 1 minute' showed lower variability among MCI patients, suggesting it’s as a precise and reliable detection tool.

Verbal memory deficits, assessed using by the short (shDFR-CVLT-II) and long (LDFR-CVLT) versions of the CVLT-II, showed significant differences in MCI [37,58,75,84]. However, only the long version (LDFR-CVLT) was meta-analyzed due to limited studies using the short version. The LDFR-CVLT demonstrated more pronounced and consistent differences with greater homogeneity, reinforcing impaired memory consolidation as an early dementia predictor. These deficits associate with hippocampal atrophy and disrupted medial temporal-prefrontal connectivity, linking structural and functional brain alterations to verbal memory impairment [28,127].

A notable finding across studies is the high prevalence of depression and anxiety in MCI [27,92-93,108]. This was consistently reported in studies that employed various emotional assessment tools, highlighting the frequent presence of affective symptoms in this population. As a result, our analysis revealed significant differences in HADS and HAM-D scores between individuals with MCI and healthy controls. While the Geriatric Depression Scale (GDS) distinguished MCI patients from healthy controls [45,65], its shorter version (GDS-short) did not detect significant differences [60]. This may reflect the greater sensitivity of the full GDS, likely due to its broader item range, which allows for a more comprehensive assessment of depressive symptoms. By contrast, the reduced number of items in the GDS-short may limit its ability to detect subtle affective changes in individuals with MCI. These findings support the use of the full GDS as a more reliable tool for identifying depression in this population.

Furthermore, we observed no significant differences in IADL measures, suggesting that daily living skills may remain relatively intact in individuals with MCI. This is consistent with the clinical definition of MCI as a condition marked by cognitive impairment without substantial functional decline. One possible explanation is that instrumental activities rely heavily on procedural memory, which is typically preserved in early MCI, unlike declarative memory. Traditional IADL scales may also lack the sensitivity to detect subtle impairments, especially in novel, complex, or cognitively demanding tasks. Given the executive and attentional deficits often observed in MCI, activities requiring multitasking, planning, or adaptability may be more affected than standard assessments reveal. Self-reported IADL measures can also be unreliable due to anosognosia, making caregiver reports or ecologically valid tools—such as digital monitoring, potentially more informative [35,97]. However, due to the high risk of bias identified in the study by Bregman et al. [97] according to the NOS scale, the reported IADL results should be interpreted with caution.

Several limitations emerged in included studies. Only approximately19% of the included studies reported MCI subtype (amnestic vs. non-amnestic), despite well-documented clinical, cognitive, and neuropathological differences between these subtypes that can materially influence performance on memory- and language-related tasks. For example, Csukly et al. [128] found distinct patterns of structural brain changes between amnestic and non-amnestic MCI. Similarly, Du et al. [129]) reported divergent regional atrophy and connectivity disruptions between the two subtypes. Neuropathological comparisons likewise reveal subtype-specific differences in underlying pathology [130]. Furthermore, HELIAD cognitive cohort has demonstrated differences in language performance metrics, with aMCI performing worse than naMCI, especially in the semantic fluency and verbal comprehension tasks [131]. The failure to report MCI subtype in the majority of included studies thus limits the interpretability and generalizability of our domain-specific findingsand precludes subgroup analyses that might clarify these patterns. We recommend that future primary studies and systematic reviews (i) report MCI subtype and (ii) perform subgroup or sensitivity analyses when ≥ 3 studies provide subtype-specific data.

In addition to subtype heterogeneity, the lack of consistent reporting on disease duration or time since symptom onset further complicates interpretation. Longitudinal studies such as those by Mitchell et al. [132] and Ward et al. [133] have shown that longer duration of MCI symptoms is associated with faster decline in memory and executive functions. Additionally, recent analyses of data from the Mayo Clinic Study of Aging (MCSA), a prospective population-based cohort with follow-up periods ranging from 30 to 105 months, utilized network analysis to explore evolving associations among clinical variables from MCI diagnosis through progression to dementia. These analyses revealed dynamic interactions between neuropsychiatric symptoms, chronic conditions, and cognitive and functional scores, with more pronounced changes observed in individuals progressing to dementia. Furthermore, sex-specific differences in clinical trajectories were noted, underscoring the complexity of disease progression over time [134]. This variability in disease duration likely reflects differences in disease stage at assessment, which may mask or exaggerate certain cognitive deficits.

Despite these challenges, this meta-analysis highlights verbal fluency, verbal memory (especially CVLT-II), and GDS as valuable early MCI indicators, supporting their use in cognitive screening. Naming tasks, however, did not consistently detect impairments, suggesting lexical access remains relatively preserved early but deteriorates with progression. This pattern aligns with gradual semantic memory decline linked to differential involvement of prefrontal and medial temporal regions. Due to inconsistent reporting, this meta-analysis cannot determine the temporal onset of memory and language impairments. The substantial heterogeneity observed in several pooled analyses (I² often > 90%) appears to be largely driven by a small number of highly influential studies with distinctive methodological and clinical characteristics. For example, Gao et al. [62] recruited a large, clinically enriched sample of MCI and subjective cognitive decline participants from a specialized memory clinic in China, applying strict inclusion and exclusion criteria and integrating neuropsychological testing with resting-state fMRI (ALFF) analyses. This high-risk, homogeneous sample, combined with imaging biomarkers, may have amplified cognitive group differences, producing larger effect sizes than those observed in community-based samples, an effect that mirrors the impact of strict recruitment and specialized interventions described in previous meta-analyses of MCI [135]. Similarly, Jung et al. [57] contributed disproportionately to heterogeneity in the CDR and IADL analyses, likely due to the inclusion of a clinically homogeneous memory clinic cohort with higher baseline functional impairment and the use of Korean-adapted versions of these scales with different sensitivity and scoring thresholds—again consistent with Huang et al.’s [135] observation that variability in diagnostic tools and outcome measures is a major source of heterogeneity. In the case of Kinsella et al. [56], the large effect size in verbal memory (CVLT-II) likely reflects the combination of an amnestic MCI sample from memory clinics, a targeted cognitive training program focusing on verbal strategies, and the use of sensitive neuropsychological measures. This aligns with Bai et al. [136], who found that even in prevalence studies of MCI, variability is strongly influenced by demographic and methodological moderators such as education, geographic region, and assessment tools.

Beyond these specific cases, other factors that may have contributed to heterogeneity in our meta-analysis, such as differences in recruitment setting, age, education, diagnostic criteria, follow-up duration, and statistical methods, were only partially assessed due to incomplete or inconsistent reporting, a limitation also highlighted by both Huang et al. [135] and Bai et al. [136] in their syntheses. As noted in these prior reviews, high heterogeneity is a common feature of MCI research, reflecting the inherent clinical and methodological diversity of the populations, diagnostic tools, and analytical approaches studied. Nevertheless, our sensitivity analyses confirmed that removing the three most influential studies substantially reduced heterogeneity (e.g., MoCA I² from 95.54% to 40.78%) while preserving the direction and, in most cases, the statistical significance of the pooled effects. This reduction to low-to-moderate heterogeneity increases confidence in the robustness of our findings, and by identifying and accounting for the main drivers of variability, our analyses help to better contextualize and interpret this heterogeneity, thereby strengthening the reliability of the conclusions.

The findings of the current review highlight the California Verbal Learning Test-II (CVLT-II) as a particularly reliable tool for memory assessment, given its consistent performance across multiple studies. Similarly, the Verbal Fluency Test (VFT) showed robust discriminative ability for MCI and should be considered a valuable tool. The Geriatric Depression Scale (GDS) also proved sensitive to affective symptoms commonly associated with MCI and may be useful in multi-domain assessments. Although WMS-Logical Memory showed promise, insufficient data prevented meta-analysis, warranting further investigation. In contrast, the Boston Naming Test (BNT) and other naming tasks proved less effective and are not recommended as primary diagnostic instruments. Lastly, while both MoCA and MMSE are commonly used, MoCA exhibited greater sensitivity to early decline, suggesting it may be better suited for early detection.

## Supplementary Materials

The Supplementary data can be found online at: www.aginganddisease.org/EN/10.14336/AD.2025.0618.
