# Supplementary material for: Neuropsychological Diagnostic Tools in Mild Cognitive Impairment: A Systematic Review and Meta-Analysis
Source: Aging Dis. 2026 Aug 20;17(6):3281–304. doi: 10.14336/AD.2025.0618 (PMC13653707; doi:10.14336/AD.2025.0618)
Supplement: Supplementary file 1 [file AD-17-6-3281-s.pdf]

SUPPLEMENTARY DATA

Neuropsychological Diagnostic Tools in Mild Cognitive Impairment: A Systematic Review and Meta-Analysis

Nuria Jerez-Garrido, Ana M. Jiménez-García, David Sánchez-Borda, Natalia Arias

© 2025. Jerez-Garrido N et al. Published online at http://www.aginganddisease.org/EN/10.14336/AD.2025.0618

SUPPLEMENTARY DATA

Supplementary Materials:

Search strategy

The search was carried out using the following terms and combinations: (mild cognitive impairment or MCI or Alzheimer's Disease or AD or Alzheim* or prodromal AD) and (Cognition or Memory or Learning or Attention or Language or Visuospatial skills or Behaviour or Mood or Anxiety or Depression) and (inventory or scale or assessment or measure or questionnaire or cognitive assessment or neuropsychological tests or psychometric tools) and (cognitive changes or cognitive improvement or cognitive decline or cognitive deterioration).

Supplementary Table 1. *PRISMA Checklist*

Section and Topic			Item #	Checklist item	Location where item is reported
TITLE					
Title			1	Identify the report as a systematic review.	Page 1
ABSTRACT					
Abstract			2	See the PRISMA 2020 for Abstracts checklist.	Page 1
INTRODUCTION					
Rationale			3	Describe the rationale for the review in the context of existing knowledge.	Page 1
Objectives			4	Provide an explicit statement of the objective(s) or question(s) the review addresses.	Page 2
METHODS					
Eligibility criteria			5	Specify the inclusion and exclusion criteria for the review and how studies were grouped for the syntheses.	Page 2-3
Information sources			6	Specify all databases, registers, websites, organisations, reference lists and other sources searched or consulted to identify studies. January 2025 was the last searched on each database.	Page 2-3
Search strategy			7	Present the full search strategies for all databases, registers and websites, including any filters and limits used.	Page 2-3
Selection process			8	Specify the methods used to decide whether a study met the inclusion criteria of the review, including how many reviewers screened each record and each report retrieved, whether they worked independently, and if applicable, details of automation tools used in the process.	Page 2-3
Data collection process			9	Specify the methods used to collect data from reports, including how many reviewers collected data from each report, whether they worked independently, any processes for obtaining or confirming data from study investigators, and if applicable, details of automation tools used in the process.	Page 3
Data items			10a	List and define all outcomes for which data were sought. Specify whether all results that were compatible with each outcome domain in each study were sought (e.g. for all measures, time points, analyses), and if not, the methods used to decide which results to collect.	Page 3
			10b	List and define all other variables for which data were sought (e.g. participant and intervention characteristics, funding sources). Describe any assumptions made about any missing or unclear information.	Page 3
Study risk of bias assessment			11	Specify the methods used to assess risk of bias in the included studies, including details of the tool(s) used, how many reviewers assessed each study and whether they worked independently, and if applicable, details of automation tools used in the process.	Page 3
Effect measures			12	Specify for each outcome the effect measure(s) (e.g. risk ratio, mean difference) used in the synthesis or presentation of results.	Page 3
Synthesis methods			13a	Describe the processes used to decide which studies were eligible for each synthesis (e.g. tabulating the study intervention characteristics and comparing against the planned groups for each synthesis (item #5)).	Page 3
			13b	Describe any methods required to prepare the data for presentation or synthesis, such as handling of missing summary statistics, or data conversions.	Page 3-4
			13c	Describe any methods used to tabulate or visually display results of individual studies and syntheses.	Page 3-4

© 2025. Jerez-Garrido N et al. Published online at http://www.aginganddisease.org/EN/10.14336/AD.2025.0618

			13d	Describe any methods used to synthesize results and provide a rationale for the choice(s). If meta-analysis was performed, describe the model(s), method(s) to identify the presence and extent of statistical heterogeneity, and software package(s) used.	Page 4-5
			13e	Describe any methods used to explore possible causes of heterogeneity among study results (e.g. subgroup analysis, meta-regression).	Page 4-5
			13f	Describe any sensitivity analyses conducted to assess robustness of the synthesized results.	Page 4-5
Reporting bias assessment			14	Describe any methods used to assess risk of bias due to missing results in a synthesis (arising from reporting biases).	Page 4-6
Certainty assessment			15	Describe any methods used to assess certainty (or confidence) in the body of evidence for an outcome.	Page 4-6
RESULTS					
Study selection			16a	Describe the results of the search and selection process, from the number of records identified in the search to the number of studies included in the review, ideally using a flow diagram.	Figure 2
			16b	Cite studies that might appear to meet the inclusion criteria, but which were excluded, and explain why they were excluded.	Page 5
Study characteristics			17	Cite each included study and present its characteristics.	Page 5
Risk of bias in studies			18	Present assessments of risk of bias for each included study.	Figure 1
Results of individual studies			19	For all outcomes, present, for each study: (a) summary statistics for each group (where appropriate) and (b) an effect estimate and its precision (e.g. confidence/credible interval), ideally using structured tables or plots.	Pages 5-15
Results of syntheses			20a	For each synthesis, briefly summarise the characteristics and risk of bias among contributing studies.	Pages 5-15
			20b	Present results of all statistical syntheses conducted. If meta-analysis was done, present for each the summary estimate and its precision (e.g. confidence/credible interval) and measures of statistical heterogeneity. If comparing groups, describe the direction of the effect.	Pages 5-15
			20c	Present results of all investigations of possible causes of heterogeneity among study results.	Pages 5-15
			20d	Present results of all sensitivity analyses conducted to assess the robustness of the synthesized results.	Pages 5-15
Reporting biases			21	Present assessments of risk of bias due to missing results (arising from reporting biases) for each synthesis assessed.	Pages 5-15
Certainty of evidence			22	Present assessments of certainty (or confidence) in the body of evidence for each outcome assessed.	Pages 5-15
DISCUSSION					
Discussion			23a	Provide a general interpretation of the results in the context of other evidence.	Page 15-18
			23b	Discuss any limitations of the evidence included in the review.	Page 16
			23c	Discuss any limitations of the review processes used.	Page 16
			23d	Discuss implications of the results for practice, policy, and future research.	Page 16-17
OTHER INFORMATION					
Registration and protocol			24a	Provide registration information for the review, including register name and registration number, or state that the review was not registered.	Page 16
			24b	Indicate where the review protocol can be accessed, or state that a protocol was not prepared.	Page 16
			24c	Describe and explain any amendments to information provided at registration or in the protocol.	Page 16
Support			25	Describe sources of financial or non-financial support for the review, and the role of the funders or sponsors in the review.	Page 18
Competing interests			26	Declare any competing interests of review authors.	Page 18
Availability of data, code and other materials			27	Report which of the following are publicly available and where they can be found: template data collection forms; data extracted from included studies; data used for all analyses; analytic code; any other materials used in the review.	Page 17-24

© 2025. Jerez-Garrido N et al. Published online at http://www.aginganddisease.org/EN/10.14336/AD.2025.0618

SUPPLEMENTARY DATA

Supplementary Table 2. Description of the studies included in the review and their reported cognitive changes.

SOURCE	PATIENTS CHARACTERISTICS	YEARS OF EDUCATION	COGNITIVE CHANGES
Bernstein et al. [80] United States	Control: n = 475 (F = 50.6%), Age = 73.07 ± 6.66 years; MCI: n = 616 (F = 40.7%), Age = 73.82 ± 7.2 years	Controls: 15.1 ± 2.5 years; MCI: 14.8 ± 3.3 years	MCI patients displayed lower results in MoCA, but higher in FAQ test than controls. No differences in GDS and GAD.
Bidzan et al. 2023 [12] Poland	MCI stable: Age = 78.79 ± 8.40 years; MCI converting: Age = 75.76 ± 9.67 years	NA	MCI stable patients displayed lower scores in MMSE but higher in CMAI than MCI converting patients.
Bowen et al. [101] United States	MCI patients: n = 17 (F = 65%), Age = 78.4 ± 8.7 years	NA	Cognitive decline measure by MoCA test correlated with lower extremity strength and sedentary behaviour.
Boyle et al [56] United States	MCI: n = 221 (F =71%, 82.8 ± 7.1 years); Controls: n = 565 (F =78.5%, 79.6 ± 6.6 years)	Controls: 14.6 ± 2.9 years; MCI: 14.3 ± 2.6 years.	Controls exhibited higher scores in MMSE than MCI patients.
Bregman et al. [98] United States	Underestimators: n = 112 (F =38.4%, 72.76 ± 7.1 years) Overestimators: n = 157 (F = 48.4%, 70.44 ± 7.4 years)	Underestimators: 16.31 ± 2.65 years; Overestimators: 16.37 ± 2.63 years	MCI patients who overestimated their performance scored higher in MMSE, GDS and ECogSubject but lower in ECogStudyPartner.
Brum et al. [105] Brazil	Study group (aMCI): n = 16 (73.3 ± 5.8 years); Control group (aMCI): n = 18 (74.0 ± 5.1 years)	aMCI study group: 9.4 ± 6.0 years; aMCI control group: 9.5 ± 5.1 years.	aMCI study and control groups scored similarly in the GDS test.
Buckley et al. [75] Australia	Controls: n = 37 (F = 35%, 77.76 ± 7.3 years); MCI: n = 43 (F = 58%, 79.6 ± 6.9 years)	NA	Controls scored lower in GDS than MCI patients.
Buerger et al. [86] Germany	Controls: n = 30 (50% female, 61.3 ± 10.2 years); MCI: n = 77 (50.64% female, 71.4 ± 7.9 years).	NA	MCI patients displayed lower in MMSE test than healthy controls.
Burggren et al. [87] United States	Controls: n = 30 (F = 50%), Age = 61.3 ± 10.2 years; MCI: n = 77 (F = 50.64%), Age = 71.4 ± 7.9 years	Controls: 16.6 ± 2.9 years; aMCI: 17.1 ± 3.3 years.	Healthy controls performed better in MMSE but lower in HAM-D than aMCI.
Calcetas et al. [88] United States	Controls: n = 20 (F = 40%), Age = 63.6 ± 11.9 years; aMCI: n = 25 (F = 65%), Age = 63.7 ± 10.7 years	Controls: 16.04 ± 2.87 years; MCI: 15.53 ± 3.10 years	No significant differences in HIS test between groups.
Capp et al. [69] Unites States	Controls: n = 27 (F = 46.4%), Age = 62.26 ± 3.9 years; aMCI: n = 21 (F = 28.6%), Age = 65.62 ± 6.7 years	Controls: 12.59 ± 1.9 years; aMCI: 12.21 ± 1.4 years	Controls displayed better results in MMSE and WRAT IV.
Cardoso et al. [70] Portugal	Controls: n = 31 (F = 77.41%), Age = 70.1 ± 7.4 years; aMCI: n = 52 (F = 57.69%), Age = 71.8 ± 8.2 years	Controls: 12.59 ± 1.9 years; aMCI: 10.4 ± 3.6 years	aMCI patients scored lower on MMSE and WMS logical memory, with no significant differences in WAIS III digit span.
Chan et al. [106] China	Stable MCI: n = 270 (F = 68.1%), Age = 76.3 ± 8.0 years Converters to dementia: n = 51 (F = 0.4%), Age = 82.7 ± 7.8 years	Stable MCI: 3.2 ± 4.0 years; Converters: 1.6 ± 3.2 years	The group that ended in dementia scored lower in MMSE and VFT.
Chang et al. [94] Taiwan	Controls: n = 18 (F =44.44%, 71.11 ± 3.55 years); MCI: n = 15 (F = 56.25%, 73.53 ± 6.39 years)	Controls: 15.44 ± 3.76 years; MCI: 12.67 ± 2.74 years.	Controls patients scored higher in MMSE, VFT-Animals and CVLT II.
Chang et al. [138] Taiwan	MCI: n = 1 (F = 100%; 30 years)	NA	She scored 10 on HAM-D and 8 on WMS-Logical Memory. She exhibited anxiety, depression, insomnia, fatigue, and reported making more mistakes than usual.

© 2025. Jerez-Garrido N et al. Published online at http://www.aginganddisease.org/EN/10.14336/AD.2025.0618

SUPPLEMENTARY DATA

Chen et al. [76] Taiwan	Controls: n = 28 (F = 57.1%, 73.7 ± 5.4 years); MCI: n = 33 (F = 69.7%, 74.9 ± 5.6 years)	Controls: 7.7 ± 5 years; MCI: 5.6 ± 4 years.	Controls displayed higher results in the CASI test.
Chen et al. [107] Taiwan	Stable aMCI: n = 13 (F = 30.8%, 73.0 ± 5.1 years) Progressed to dementia: n = 9 (F = 55.6%, 76.0 ± 2.77 years)	Stable group: 12.0 ± 2.37 years; Group that ended in dementia: 12.0 ± 2.83 years.	The stable group obtained higher scores in MMSE and CVLT II.
Cook et al. [71] United States	Controls: n = 54 (F = 64.8%, 74.3 ± 5.0 years); aMCI: n = 17 (F = 70.6%, 77.0 ± 7.4 years)	Controls: 16.1 ± 2.6 years; aMCI: 16.29 ± 3.1 years.	Control subjects scored higher in MMSE but lower in GDS than aMCI patients.
De Anna et al. [78] France	Controls: n = 26 (F = 46.15%), Age = 67.5 ± 7.4 years<br>aMCI – better DMS48: n = 15 (F = 46.6%), Age = 72.06 ± 7.0 years<br>aMCI – worse DMS48: n = 18 (F = 38.88%), Age = 67.5 ± 7.1 years	Controls: 11.6 ± 3.1 years; aMCI with better scores in DMS48: 12.4 ± 4.3 years; aMCI with worse scores in DMS48: 12 ± 4.5 years.	Controls scored higher in FCSRT delayed and total recall, and MMSE, but lower in IADL. aMCI patients with better DMS48 scores performed better than those with worse DMS48 scores.
de Mendonça et al. [95] Portugal	MCI patients: n = 7 (F = 57.1%; 58.57 ± 12.98 years)	NA	Impaired attention (patients 1, 3, 5, 6), memory deficits (1, 2, 3, 6), and graphomotor initiative problems (most patients). Motor and constructional abilities were reduced in some cases (1, 3, 4), and learning/abstraction was impaired in a few (1, 6).<br>Behavioral changes included irritability (2, 3, 5, 7), loss of initiative (2, 3), impulsivity and disinhibition (6), perseveration (7), childish behavior (3), obsessive focus on minor problems (1), and emotional instability (2, 7). Daily living activities were generally preserved, with some exceptions (financial mismanagement in patient 4).
Devanand et al. [73] United States	Controls: n = 57 (F = 56%; 65.9 ± 9.4 years)<br>MCI: n = 136 (F = 55%; 67.1 ± 9.9 years)	Controls: 16.8 ± 2.6 years; MCI: 15 ± 4.3 years.	Although healthy controls scored higher in MMSE, they scored lower in FCSRT delayed recall.
Eramudugolla et al. [67] Australia	Controls: n = 242 (F = 40.9%; 75.00 ± 2.00 years); MCI: n = 54 (F = 37.8%; 75.00 ± 2.25 years)	Control 16 ± 1,25 years of education while MCI had 16 ± 1 years.	Both, controls and MCI scored similar in MMSE test.
Ewers et al. [72] United States	Controls: n = 100 (F = 55%), Age = 72.8 ± 5.36 years<br>MCI: n = 184 (F = 41.85%), Age = 72.9 ± 7.11 years	Controls 16.1 ± 2.8 years; MCI 16.1 ± 2.81 years.	MCI subjects displayed higher results in ADAS Cog13 than controls.
Facal et al [74] Spain	Controls: n = 58 (67.36 ± 9.09 years); Single-domain aMCI: n = 43 (67.62 ± 9.40 years); Multiple-domain aMCI: n = 44 (70.34 ± 9.49 years)	Controls: 10.22 ± 5.05 years; Single domain aMCI: 9.23 ± 4.1 years; Multiple domain aMCI: 9.54 ± 3.77 years.	aMCI patients (single and multiple domain) scored lower than controls on several tests, with no significant MMSE differences between single domain aMCI and healthy controls.
Faria et al. [66] Brazil	Stable MCI: n = 4 (F = 100%; 78.0 ± 6.27 years)<br>Progressing to dementia: n = 9 (F = 66.7%; 76.44 ± 8.81 years)	MCI: 8.50 ± 6.75 years; Dementia: 5.33 ± 5.24 years.	The MCI stable group scored lower in ADL but higher in GDS than the MCI patients who ended in dementia.
Fodor et al. [65] Hungary	Controls: n = 21 (F = 66.7%), Age = 64.9 ± 5.2 years; MCI: n = 17 (F = 47%), Age = 69.2 ± 7 years	NA	Healthy controls scored higher across RAVLT delayed recall, ACE, MMSE and STAI than MCI patients. There were no significant differences in GDS, TMT A and TMT B scores.
Forstmeier et al. [64] Germany	Controls: n = 1748 (F = 65.7%; 79.3 ± 3.2 years); MCI: n = 313 (F = 64.9%; 79.9 ± 3.6 years)	Controls: 11.9 ± 2.1 years; MCI: 12.5 ± 2.7 years.	Healthy controls displayed better results in MMSE. In ADL differences were not significant.<br>Motivation-related occupational abilities were associated with a reduced risk of MCI.

© 2025. Jerez-Garrido N et al. Published online at http://www.aginganddisease.org/EN/10.14336/AD.2025.0618

SUPPLEMENTARY DATA

Gao et al. [63] Finland	Controls: n = 36 (F = 41.7%), Age = 71.72 ± 6.49 years; MCI: n = 44 (F = 54.5%), Age = 69.46 ± 7.22 years	Controls: 12 years; MCI: 12 years	MCI patients displayed lower scores in MMSE, MoCA, BNT, VFT-Animals, WMS, Digit Span and SDM than healthy controls. In TMT A and TMT B their scores were higher.
Gerstenecker et al. [108] United States	MCI: n = 52 (F = 46.15%; 71.0 ± 17.68 years)	MCI: 14.90 ± 7.25 years.	They obtained low scores in MMSE, digit span and CVLT II, but high in CDR, TMT A and TMT B.
Ghoshal et al. [62] United States	Controls: n = 12 (F = 58%), Age = 79.8 ± 7 years; MCI: n = 12 (F = 42%), Age = 85.3 ± 6.3 years	Controls: 17.8 ± 3.3 years; MCI: 17.3 ± 4.1 years	Controls scored higher across all tests than MCI patients in MMSE, WMAE logical memory I and II.
Gonzales et al. [109] United States	MCI without SSD: n = 69 (F = 31.88%; 73.71 ± 7.99 years); MCI with SSD: n = 32 (F = 25%; 75.84 ± 7.59 years)	MCI without SSD: 15.93 ± 3.01 years; SSD group: 15.62 ± 2.86 years. The SSD group displayed accelerated decline in global cognition.	MCI without SSD scored significantly lower in GDS and NPI than MCI patients with SSD. No differences were found in MMSE.
Grundman et al. [79] United States	Controls: n = 107 (F = 59.6%), Age = 70 ± 8.3 years; MCI: n = 769 (F = 45.8%), Age = 72.9 ± 7.3 years	Controls: 14.8 ± 3.3 years; MCI: 14.7 ± 3.1 years	Healthy controls scored higher in MMSE; whereas they scored lower in CDR, HAM-D and HIS than MCI patients.
Gugała-Iwaniuk, et al. [61] Poland	Controls: n = 33 (F = 60.60%; 67.66 ± 6.41 years); naMCI: n = 35 (F = 60%; 65.68 ± 6.88 years); aMCI: n = 15 (F = 86.66%; 69.20 ± 6.68 years).	Controls: 13.6 ± 3.13 years; naMCI: 16 ± 3 years; aMCI: 12.8 ± 2.81 years.	Differences in GDS between healthy controls, naMCI and aMCI groups were not significant.
Hansen et al. [6] United States	aMCI (longitudinal data): n = 52 (F = 48%), Age = 76.17 ± 5.62 years; aMCI (clinical settings): n = 53 (F = 51%), Age = 73.09 ± 6.60 years	aMCI detected in longitudinal data: 14.92 ± 3.04 years; aMCI detected in clinical setting: 15.79 ± 2.87 years.	aMCI patients detected in longitudinal data displayed higher results across all tests except TMT A, including CFT copu, AVLT-TL, AVLT-STM, AVLT-LTM, AVLT-recog, AVLT-FP, BNT, DRS, VFT-Animals and TMT B.
Hanyu et al. [112] Japan	Early MCI: n = 14 (F = 50%; 74.9 ± 6.6 years); LMCI−: n = 20 (F = 50%; 73.5 ± 8.0 years); LMCI+: n = 37 (F = 64.9%; 74.7 ± 6.5 years)	Early MCI: 13.2 ± 2.3 years; LMCI-: 14.5 ± 2.4 years; LMCI+: 14.1 ± 2.4 years. Path integration performance was lowest in LMCI+.	LMCI- patients scored the lowest in MMSE and MoCA, followed by LMCI+. Early MCI patients scored lowest in GDS-15, while LMCI- scored the highest.
Headley et al. [60] United States	Controls: n = 111 (F = 50%; 75 ± 6 years); MCI: n = 193 (F = 33%; 75 ± 7 years).	Controls: 16 ± 3 years; MCI: 16 ± 3 years.	MCI patients scored higher in CDR but lower in MMSE.
Hirao et al. [110] Japan	aMCI: n = 24 (F = 37,5%; 76,7 ± 5,8 years)	aMCI patients stated 13,5 ± 2,6 years of education.	aMCI obtained low scores in MMSE and VFT, but higher in TMT A and TMT B.
Jang et al. [111] South Korea	MCI: n = 5 (66,6 ± 3,5 years)	MCI patients had 9,2 ± 3,6 years of education.	Low scores in the MoCA test.
Johansson et al. [93] Sweden	Controls: n = 104, F = 50%, 72,1 ± 5,3 years; MCI: n = 53, F = 41,5%, 70,4 ± 5,7 years	Controls: 11.9 ± 3.1 years; MCI: 11.2 ± 3.6 years.	Healthy controls scored higher than MCI patients on MMSE, but lower on ADAS-Cog, HADS-D, and HADS-A.
Jongbloed et al. [59] Netherlands	naMCI: n = 31, F = 41,93%, 69 ± 9 years; aMCI: n = 19, F = 36,84%, 68 ± 9 years; Controls: n = 67, F = 64,17%, 64 ± 8 years	NA	Healthy controls displayed higher results than aMCI and naMCI in MMSE. Differences between MCI groups were not significant.
Jung et al. [58] South Korea	Controls: n = 24 (F = 51%; 73.6 ± 0.5 years); MCI: n = 18 (F = 69.7%; 75.9 ± 0.5 years)	NA	Controls scored higher than MCI patients on MMSE, but lower on CDR, IADL, and GDS.
Kang et al. [113] South Korea	MCI: n = 98, F = 77,6%, 74,7 ± 5,39 years	MCI: 5,4 ± 4,9 years.	MCI patients scored 4,0 ± 3,9 in IADL test.

© 2025. Jerez-Garrido N et al. Published online at http://www.aginganddisease.org/EN/10.14336/AD.2025.0618

Kashimura et al. [139] Japan	Single patient: 70 years old, female (F = 100%).	NA	She obtained 28 in MMSE; 10 in GDS-15 and 9 in HADS-A.
Kinsella et al. [57] Australia	Controls: n = 113 (F = 74%), Age = 72.27 ± 6.95 years; aMCI: n = 106 (F = 58%), Age = 76.08 ± 7.1 years	NA	aMCI patients scored lower than controls across MMSE, HVLT delayed recall, WMS logical memory delayed, WMS IV verbal paired associates, CVLT II and RFDR.
Kirsebom et al. [55] Norway	Controls: n = 20 (F = 50%), Age = 62.8 ± 9.6 years; MCI: n = 20 (F = 57%), Age = 66.8 ± 7.4 years	NA	MCI displayed lower results than controls across all tests, including: MMSE, CERAD learning, CERAD recall, TMT A and TMT B.
Kivisäkk et al. [54] United States	Controls: n = 90 (F = 56.7%), Age = 72.6 ± 10.4 years; naMCI: n = 38 (F = 44.7%), Age = 77.8 ± 7.4 years; aMCI: n = 47 (F = 48.9%), Age = 75.7 ± 8.6 years	NA	aMCI and naMCI scored lower in MMSE but higher in CDR than controls. Differences between naMCI and aMCI were not significant.
Klekociuk and Summers [92] Australia	50 controls (72,66 ± 6,52 years); 23 aMCI patients (70,61 ± 7,99 years) and 26 naMCI patients (70,58 ± 5,97 years).	Controls: 14.20 ± 3.74 years; aMCI: 14.43 ± 3.16 years; naMCI: 14.92 ± 3.52 years.	Controls, aMCI and naMCI patients did not exhibit significant differences across HADS-A and HADS-D tests.
Kuhn et al. [53] France	Controls: n = 32 (F = 50%), Age = 70.91 ± 6.57 years; aMCI: n = 35 (F = 34%), Age = 72.49 ± 7.5 years	aMCI: 11.46 ± 3.86 years; controls: 12.22 ± 3.68 years.	aMCI patients scored higher in I-SMD but lower in MMSE compared to healthy controls.
Lai et al. [52] Taiwan	Controls: n = 14 (F = 35.71%), Age = 64.79 ± 7.75 years; aMCI: n = 18 (F = 63.63%), Age = 68 ± 8.7 years	Controls 9.7 ± 4.16 years; aMCI 9.89 ± 5.16 years.	aMCI patients showed lower scores in MMSE and CASI score than healthy patients.
Landau et al. [81] United States	Controls: n = 126 (F = 50%), Age = 78.4 ± 5.8 years; Early MCI: n = 162 (F = 43%), Age = 71.2 ± 7.6 years; Late MCI: n = 85 (F = 37%), Age = 76.3 ± 8.8 years	Controls 16.4 ± 2.8 years, early MCI 15.9 ± 2.6, late MCI 15.9 ± 3.2.	Controls scored better in MMSE but worse in ADAS Cog than MCI patients, with early MCI outperforming late MCI in MMSE and underperforming in ADAS Cog.
Lazarou et al. [51] Greece	Controls: n = 65, Age = 62.94 ± 7.6 years; MCI: n = 89, Age = 70.96 ± 7.87 years	Controls: 14.77 ± 2.99 years; MCI: 14.33 ± 2.55 years.	Healthy controls exhibited better results in MMSE tests than MCI patients.
Lazarou et al. [50] Greece	MCI: n = 23 (F = 69.56%), Age = 69.61 ± 6.91 years; Controls: n = 21 (F = 61.90%), Age = 62.62 ± 13.86 years	Controls: 14.7 ± 5.32 years; MCI: 10.08 ± 4.36 years	MCI patients scored lower than healthy controls on MMSE, NPI, FRSSD, TMT B, RBMT immediate and delayed recall, and VFT, but higher in FUCAS and ROCFT copy.
Lim et al. [49] Australia	PiB+ Controls: n = 55 (F = 51%), Age = 75.2 ± 7.19 years; PiB− Controls: n = 22 (F = 49%), Age = 74.39 ± 7.34 years; PiB+ MCI: n = 32 (F = 52%), Age = 75.76 ± 5.89 years; PiB− MCI: n = 16 (F = 50%), Age = 75.56 ± 9.68 years	NA	Controls exhibited higher results in MMSE test but lower in HADS-D and HADS-A than aMCI patients. Differences between aMCI groups were not significant.
Lim et al. [48] Australia	PiB+ controls (n = 55, F = 51%, 75,2 ± 7,19 years), PiB− controls (n = 22, F = 49%, 74,39 ± 7,34 years), PiB+ MCI (n = 32, F = 52%, 75,76 ± 5,89 years), and PiB− MCI (n = 16, F = 50%, 75,56 ± 9,68 years).	NA	MCI patients scored higher across all tests than controls independently of their PiB (a ligand for labelling amyloid), which performed similarly in CDR, HADS-D and HADS-A.
Lin et al. [114] Australia	aMCI: n = 49 (F = 38.8%), Age = 68.31 ± 9.25 years; naMCI: n = 99 (F = 66.67%), Age = 67.72 ± 8.77 years	aMCI: 13.72 ± 3.08 years; naMCI: 13.81 ± 3.35 years.	naMCI patients scored higher in MMSE, HAM-D and HADS-D than aMCI patients. In HADS-A differences were not significant.
Lopes et al. [47] Brazil	MCI: n = 29 (F = 79.3%), Age = 75 ± 5.6 years; Controls: n = 24 (F = 75%), Age = 70.3 ± 5.4 years	Controls: 5.7 ± 3.5 years; MCI: 5.4 ± 5.5 years	MCI patients scored lower in MMSE and CAMCOG.

© 2025. Jerez-Garrido N et al. Published online at http://www.aginganddisease.org/EN/10.14336/AD.2025.0618

MacAulay et al. [46] United States	Controls: n = 27 (F = 51.9%), Age = 63.3 ± 9.89 years; aMCI: n = 34 (F = 47.1%), Age = 73.06 ± 8.14 years	Controls: 14.62 ± 2.95 years; MCI: 14.26 ± 2.42 years.	Healthy controls scored higher in MMSE test than MCI patients.
Maeng et al. [45] South Korea	Controls: n = 25 (F = 88%; 71.6 ± 4.4 years); MCI: n = 31 (F = 74.2%; 73.2 ± 7.3 years). Participants underwent a fully immersive virtual reality cognitive training program for 4 weeks.	Controls: 8.9 ± 3.4 years; MCI: 9.5 ± 4.7 years.	MCI patients exhibited lower results in Quality of Life-AD and higher in GDS than controls.
Messinis et al. [115] Greece	MCI: n = 76 (75 ± 1,75 years; F = 70%).	MCI: 12 ± 2 years.	They scored 27,5 ± 0,75 in MMSE test.
Metzler-Baddeley et al. [44] United Kingdom	AMCI: n = 25 (F = 56%; 76,8 ± 7,3 years) in contrast with 20 controls (F = 50%; 74 ± 6,5 years).	aMCI: 14 ± 3.7 years; Controls: 15 ± 2.8 years	Healthy controls scored higher than aMCI patients on all tests (WAIS III symbol substitution, VFT, Category Fluency, and NART).
Mofrad et al. [84] United States	Controls: n = 134, F = 100%, 77,2 ± 7 years; naMCI: n = 333, F = 100%, 72,6 ± 6 years.	Controls: 16.1 ± 3 years; naMCI: 13.1 ± 3 years.	Healthy controls outperformed naMCI patients on RAVLT immediate, with no significant differences in ADAS 13 scores..
Montero-Odasso et al. [91] Canada	Controls: n = 35 (F = 82.86%), Age = 70.37 ± 3.93 years; naMCI: n = 22 (F = 63.68%), Age = 74.18 ± 6.54 years; aMCI: n = 42 (F = not reported), Age = 77.33 ± 7.26 years,	NA	Controls obtained the highest scores across MMSE and MoCA, followed by the naMCI group.
Moscoso et al. [82] Spain	Controls: n = 113 (F = 50%), Age = 75 ± 6 years; Early MCI: n = 132 (F = 38%), Age = 73 ± 7 years; Late MCI: n = 255 (F = 37%), Age = 74 ± 7 years.	Controls: 16 ± 3 years; Early MCI: 16 ± 3 years; Late MCI: 16 ± 3 years.	Healthy controls exhibited higher scores in MMSE and lower in CDR than MCI patients. Between the MCI groups, early MCI performed better in MMSE and worse in CDR.
Mura et al. [116] France	MCI: n = 212, F = 59%, 71,8 ± 5,3 years.	MCI: 7.47 ± 4.15 years	Most sensitive tests evaluating cognitive changes were FCSRT, VFT and Deno100.
Papaliagkas et al. [140] Greece	MCI: n = 1, F = 0%, 74 years.	MCI: 6 years	He scored 27/30 on MMSE, 0.5 on CDR, and 0/15 on GDS.
Park [43] South Korea	MCI: n = 76 (F = 40.8%), Age = 66.5 ± 9.2 years; Controls: n = 76 (F = 40.8%), Age = 66.7 ± 9.2 years	MCI: 17.9 ± 4.2 years; Controls: 8 ± 4.3 years.	MCI patients scored lower than controls on MMSE and CDR, but higher on IQCODE.
Perrotin et al. [42] Canada	aMCI: n = 20, F = 50%, 67,45 ± 8,42 years; Controls: n = 20, F = 75%, 68,55 ± 7,84 years	Controls: 14.4 ± 3.28 years; aMCI: 14.65 ± 3.7 years.	aMCI patients scored lower than healthy controls on MDRS, with no significant differences in MMSE and GDS.
Polcher et al. [41] Germany	MCI: n = 29 (F = 51.72%; 68.21 ± 8.97 years); Controls: n = 28 (F = 42.85%; 67.46 ± 8.12 years)	Controls: 14.54 ± 3.12 years; MCI: 14.07 ± 2.94 years.	MCI patients had higher scores in GDS than controls but lower in CERAD.
Poptsi et al. [117] Greece	MCI: n = 245, F = 76,73%, 67,39 ± 8,80 years	MCI: 10.88 ± 4.76 years	MoCA: 22.75 ± 3.60.
Rabin et al. [40] United States	aMCI: n = 18 (F = 61.1%; 81.56 ± 6.98 years); naMCI: n = 38 (F = 81.57%; 80.42 ± 5.72 years): Controls: n = 118 (F = 65%; 80.31 ± 5.52 years)	Controls: 16.06 ± 2.86 years; aMCI: 13.56 ± 4.44 years; naMCI: 11.84 ± 2.91 years.	aMCI and naMCI scored lower than healthy controls in GDS but higher in RPA-ProMem and FCSRT, with the difference in FCSRT not being statistically significant in the naMCI group.
Ramakers et al. [18] Netherlands	MCI: n = 343, F = 38%, 60,9 ± 9,9 years	MCI: 12,56 ± 2,77 years.	They scored 28,1 ± 1,8 in MMSE and 2,4 ± 0,5 in GDS.

© 2025. Jerez-Garrido N et al. Published online at http://www.aginganddisease.org/EN/10.14336/AD.2025.0618

SUPPLEMENTARY DATA

Richard et al. [119] United States	MCI without apathy or depressed affect: n = 92, F = 36,9%, 75,9 ± 6,6 years; MCI with apathy only: n = 59, F = 36,6%, 77,5 ± 7,2 years; MCI with depressed affect only: n = 127, F = 37%, 72,8 ± 7,5 years; MCI with both apathy and depressed affect: n = 119, F = 34,6%, 74,7 ± 7,7 years	MCI with no symptoms of apathy or depressed affect: 15.7 ± 3.0 years; MCI with apathy symptoms only: 15.6 ± 2.8 years; MCI with depressed affect symptoms only: 15.8 ± 3.0 years; MCI with both apathy and depressed affect symptoms: 15.6 ± 3.2 years.	MCI patients with no symptoms of apathy or depressed affect scored the highest in MMSE, followed by MCI patients with apathy symptoms only, MCI patients with symptoms of depressed affect only, and finally MCI patients with symptoms of both apathy and depressed affect.
Riemenschneider et al. [120] Germany and Australia	Progressive MCI: n = 6 (F = 50%; 65.7 ± 8.4 years); Stable MCI: n = 10 (F = 40%; 67.5 ± 10.7 years)	NA	Progressive MCI patients scored lower across all tests (MMSE, CERAD delayed verbal recall and GDS).
Rodakowski et al. [39] United States	Controls: n = 96 (F = 79.2%; 72.5 ± 5.9 years); aMCI: n = 61 (F = 78.7%; 75.5 ± 6.3 years)	Controls: 16.06 ± 2.86 years; MCI: 12.9 ± 2.3 years.	MCI patients displayed higher scores in HAM-D and IADL tests, but lower in MMSE than controls.
Schreiber et al. [121] United States	MCI Aβ-: n = 107 (F = 59.3%), Age = 73.5 ± 7.6 years MCI Aβ+: n = 187 (F = 59.5%), Age = 74.8 ± 6.4 years	MCI Aβ-: 16.2 ± 2.6 years; MCI Aβ+: 16.1 ± 2.8 years.	MCI Aβ- patients had lower ADAS Cog score but higher RAVLT score than MCI Aβ+ patients.
Segkouli et al. [68] Greece	Controls: n = 10 (F = 40%; 66.2 ± 2.89 years); aMCI: n = 8 (F = 50%; 67.38 ± 7.81 years)	NA	aMCI patients scored lower in BNT than controls.
Small et al. [90] United States	Controls: n = 22 (F = 64%; 65.5 ± 13.28 years); aMCI: n = 21 (F = 57%; 64.0 ± 12.7 years)	Healthy controls: 17 ± 2.89 years; aMCI: 16 ± 3.46 years.	Controls and aMCI patients scored similarly in MMSE, HAM-D, and ROCFT copy. Controls outperformed aMCI in RFDR, BNT, VFT-Animals, WAIS III digit symbol, and Stroop color, while aMCI performed better in TMT A and B.
Snider et al. [122] United States	MCI: n = 49, F = 38,77%, 73,8 ± 10,0 years	MCI: 15,2 ± 3,1 years	MMSE: 26,4 ± 2,9.
Stewart et al. [97] United States	MCI: n = 15 (F = 80%), Age = 71.3 ± 2.4 years; Control: n = 20 (F = 55%), Age = 68.9 ± 1.9 years	Controls: 15 ± 0.5 years; MCI: 15.9 ± 0.5 years.	Healthy controls outperformed MCI patients in MMSE, DRS-total, and DRS-memory, with no significant differences in DRS-attention.
Sugimoto et al. [123] Japan	MCI: n = 173, F = 56,7%, 77,9 ± 4,8 years	MCI: 11,6 ± 2,7 years	They scored 25,2 ± 2,4 in MMSE and 2,9 ± 2,4 in GDS-15.
Taler et al. [38] Canada	aMCI: n = 18 (F = 27.77%), Age = 75.56 ± 6.59 years; Control: n = 18 (F = 50%), Age = 69.28 ± 4.66 years	aMCI: 16.33 ± 3.36 years; Controls: 16.06 ± 2.86 years.	aMCI patients scored lower than healthy controls in MoCA, WCST, WMS-R immediate and delayed, BNT, Letter Fluency, and VFT-Animals.
Thomas et al. [37] United States	Patients: n = 475 (F = 50.6%), Age = 73.07 ± 6.66 years; MCI: n = 616 (F = 40.7%), Age = 73.82 ± 7.2 years	Controls: 16.4 ± 2.73 years; MCI: 15.81 ± 2.87 years.	Healthy controls scored higher than MCI patients in MMSE, while MCI patients scored higher in GDS.
Toth et al. [36] Hungary	MCI: n = 48 (F = 66.66%), Age = 73.08 ± 7.95 years; Controls: n = 36 (F = 63.88%), Age = 64.13 ± 7.08 years	MCI: 11.82 ± 3.29 years; Controls: 12.47 ± 3.21 years.	MCI patients exhibited lower results in MMSE but higher results in the ADAS Cog than healthy controls.
Traykov et al. [89] France	Controls: n = 20 (F = 30%), Age = 73.3 ± 7.0 years; MCI: n = 20 (F = 20%), Age = 73.2 ± 8.0 years	Controls: 12.8 ± 3.3 years; MCI: 12.1 ± 3.1 years.	Healthy controls scored higher than MCI patients in MMSE, while MCI patients scored higher in GDS.

© 2025. Jerez-Garrido N et al. Published online at http://www.aginganddisease.org/EN/10.14336/AD.2025.0618

Tröger et al. [127] Netherlands	MCI: n = 8, F = 25%, 68,63 ± 8,81 years	NA	CDR: 0.44 ± 0.18; MMSE: 26.88 ± 2.17.
Troyer and Murphy [35] United States	aMCI: n = 45 (F = 47%), Age = 75.8 ± 6.7 years; Controls: n = 42 (F = 59%), Age = 75.1 ± 5.7 years	Healthy controls: 13.8 ± 3.3 years; aMCI: 13.6 ± 3.3 years.	aMCI patients scored lower than healthy controls in MMSE, HVLT-R immediate recall, and TMT B, but higher in WAIS III Digit Span.
Troyer et al. [34] United Kingdom	aMCI: n = 29 (F = 55%), Age = 75.1 ± 7.1 years; Controls: n = 30 (F = 57%), Age = 75.2 ± 5.7 years	Healthy controls: 14.1 ± 3.3 years; aMCI: 14.3 ± 2.6 years.	Healthy controls outperformed aMCI patients in most tests, except for WAIS III digit span.
Tsunoda et al. [33] Japan	MCI: n = 146 (F = 52.05%), Age = 76.9 ± 8.2 years; Controls: n = 218 (F = 59.63%), Age = 71.7 ± 8.9 years	NA	MCI portrayed lower MMSE scores but higher CDR, ABS and MBI than healthy controls.
Tu et al. [124] Taiwan	MCI: n = 15 (F = 53,33%; 61,7 ± 7,75 years).	MCI: 9,0 ± 3,4 years.	MMSE: 28.2 ± 1.58; CDR: 1.0 ± 0.88; Digit span: 19.4 ± 4.03.
Van der Flier et al. [32] Netherlands	Controls: n = 28 (F = 42.85%), Age = 74 ± 7 years; MCI: n = 13 (F = 23.07%), Age = 74 ± 8 years	MCI: 10 ± 3 years; Controls: 9 ± 3 years.	MCI patients had lower scores in the MMSE than healthy controls.
Van Patten et al. [31] United States	MCI: n = 206 (F = 54.9%), Age = 74.88 ± 7.54 years Controls: n = 87 (F = 51.7%), Age = 69.71 ± 7.61 years	Healthy controls: 15.21 ± 2.69 years; MCI: 14.17 ± 3.06 years.	aMCI and naMCI were assembled in a single group and performed MMSE and 3MS with lower results than healthy patients.
Visser et al. [30] Europe	naMCI: n = 37 (F = 46%), Age = 70.0 ± 7.7 years; aMCI: n = 37 (F = 48%), Age = 70.0 ± 7.7 years; Controls: n = 89 (F = 54%), Age = 67.1 ± 6.4 years	naMCI: 10.7 ± 3.7 years; aMCI: 10.4 ± 3.06 years.	naMCI patients presented lower scores in MMSE than controls. However, aMCI patients obtained the worst results.
Wang et al. [125] United States	Stable aMCI: n = 155, F = 47%, 70 ± 7 years; Mild decline aMCI: n = 336, F = 42%, 74 ± 8 years; Moderate decline aMCI: n = 240, F = 42%, 74 ± 7 years; Aggressive decline aMCI: n = 105, F = 43%, 74 ± 7 years	Stable aMCI: 17 ± 2 years; Mild decline aMCI: 16 ± 3 years; Moderate decline aMCI: 16 ± 3 years; Aggressive decline aMCI: 15 ± 3 years.	Stable aMCI patients scored the highest in MMSE and lowest in ADAS Cog, followed by mild decline aMCI, moderate decline aMCI and aggressive decline aMCI.
Wei et al. [126] United States	Early MCI: n = 100 (F = 40%), Age = 73.09 ± 6.70 years; Late MCI: n = 97 (F = 49.45%), Age = 72.54 ± 6.88 years	Early MCI: 15.70 ± 2.844 years; Late MCI: 16.58 ± 2.657 years.	Early MCI patients outperformed late MCI patients in MMSE, MoCA, RAVLT learning and forgetting, BNT, and VFT, but scored lower in CDR, ADAS, RAVLT immediate and forgetting, EcogPtTotal, TMT A, and TMT B.
Weitzner and Calamia [85] United States	Controls: n = 57 (F = 78.9%; 71.86 ± 7.11 years); MCI: n = 29 (F = 51.7%; 71.14 ± 7.47 years)	Controls: 16.28 ± 2.48 years; MCI: 16.21 ± 1.95 years.	Healthy controls scored higher in NAB, Category Fluency, Letter Fluency, CVLT; ShDRF and LDFR than MCI patients.
Wolk et al. [29] United States	Controls: n = 50 (F = 58%; 71.20 ± 9.0 years); aMCI: n = 32 (F = 53.1%; 72.0 ± 6.9 years)	Controls: 16.20 ± 2.80 years; aMCI: 16.90 ± 2.30 years.	aMCI scored lower than controls in MMSE, CERAD tests, Digit Span Forward, VFT-Animals, and BNT-30, but similar in Digit Span backward and higher in TMT A and B.
Xie et al. [28] United States	Controls: n = 25 (F = 48%; 74.28 ± 8.25 years); aMCI: n = 17 (F = 64.7%; 75.12 ± 6.62 years)	Controls: 15.32 ± 2.87 years; aMCI: 13.47 ± 2.07 years.	aMCI scored lower than healthy controls in MMSE, DRS-Attention, DRS-Memory, DRS-Total, WMS Logical Memory Immediate and Delayed, HAM-Anxiety, and GDS.
Xu et al. [104] China	Low CRP MCI: n = 126 (F = 32%), Age = 69.5 ± 7.9 years; High CRP MCI: n = 42 (F = 23.8%), Age = 71.5 ± 6.3 years	Low plasma CRP: 4.9 ± 2.3 years; MCI with high plasma CRP: 5.2 ± 2.5 years.	MCI patients with low plasma CRP obtained higher results than MCI patients with high plasma CRP in MMSE test.
Yokoi et al. [103] Japan	Converters to AD: n = 101 (F = 60.4%; 73.08 ± 5.63 years); Non-converters: n = 133 (F = 42.9%; 72.55 ± 6.07 years)	MCI converters to AD: 12.61 ± 2.93 years; MCI non-converters: 13.32 ± 2.71 years.	MCI patients who converted to AD scored higher than non-converters in GDS, ADAS-Cog, and CDR, but lower in HIS and MMSE.
Zandifar et al. [102]	aMCI: n = 31, F = 34%, 73 ± 7 years	aMCI: 16 ± 2.55 years.	aMCI patients obtained a score of 28 ± 1 in the MMSE test.

© 2025. Jerez-Garrido N et al. Published online at http://www.aginganddisease.org/EN/10.14336/AD.2025.0618

United States			
Zhang et al. [99] China	MCI Mahjong players: n = 35 (F = 78.6%), Age = 74.4 ± 3.9 years; MCI non-players: n = 34 (F = 67.9%), Age = 74.2 ± 4.8 years	MCI Mahjong: 5.6 ± 3.7 years; MCI non-players: 5.9 ± 4.2 years.	No differences were found in GDS and CDR, but significant improvements were observed in MoCA and FAQ tests for MCI patients who played Mahjong.
Zhu et al. [100] China	MCI: n = 126 (F = 39.68%), Age = 73.3 ± 7.6 years	MCI: 15.9 ± 2.7 years.	MCI patients scored low in MMSE, MoCA, BNT and RAVLT delayed recall, but high in TMT A and B.

© 2025. Jerez-Garrido N et al. Published online at http://www.aginganddisease.org/EN/10.14336/AD.2025.0618
